# Telomere-to-telomere gap-free genome assembly provides genetic insight into the triterpenoid saponins biosynthesis in *Platycodon grandiflorus*

**DOI:** 10.1093/hr/uhaf030

**Published:** 2025-02-01

**Authors:** Hanwen Yu, Haixia Wang, Xiao Liang, Juan Liu, Chao Jiang, Xiulian Chi, Nannan Zhi, Ping Su, Liangping Zha, Shuangying Gui

**Affiliations:** College of Pharmacy, Anhui University of Chinese Medicine, Hefei 230012, China; College of Pharmacy, Anhui University of Chinese Medicine, Hefei 230012, China; College of Pharmacy, Anhui University of Chinese Medicine, Hefei 230012, China; State Key Laboratory for Quality Ensurance and Sustainable Use of Dao-di Herbs, National Resource Center for Chinese Materia Medica, China Academy of Chinese Medical Sciences, Beijing 100700, China; State Key Laboratory for Quality Ensurance and Sustainable Use of Dao-di Herbs, National Resource Center for Chinese Materia Medica, China Academy of Chinese Medical Sciences, Beijing 100700, China; State Key Laboratory for Quality Ensurance and Sustainable Use of Dao-di Herbs, National Resource Center for Chinese Materia Medica, China Academy of Chinese Medical Sciences, Beijing 100700, China; College of Pharmacy, Anhui University of Chinese Medicine, Hefei 230012, China; State Key Laboratory for Quality Ensurance and Sustainable Use of Dao-di Herbs, National Resource Center for Chinese Materia Medica, China Academy of Chinese Medical Sciences, Beijing 100700, China; College of Pharmacy, Anhui University of Chinese Medicine, Hefei 230012, China; Institute of Conservation and Development of Traditional Chinese Medicine Resources, Anhui Academy of Chinese Medicine, Hefei 230012, China; MOE-Anhui Joint Collaborative Innovation Center for Quality Improvement of Anhui Genuine Chinese Medicinal Materials, Hefei 230012, China; Center for Xin'an Medicine and Modernization of Traditional Chinese Medicine of IHM, Anhui University of Chinese Medicine, Hefei 230012, China; College of Pharmacy, Anhui University of Chinese Medicine, Hefei 230012, China; MOE-Anhui Joint Collaborative Innovation Center for Quality Improvement of Anhui Genuine Chinese Medicinal Materials, Hefei 230012, China; Institute of Pharmaceutics, Anhui Academy of Chinese Medicine, Hefei 230012, China; Anhui Province Key Laboratory of Pharmaceutical Preparation Technology and Application, Hefei 230012, China

## Abstract

*Platycodon grandiflorus* has been widely used in Asia as a medicinal herb and food because of its anti-inflammatory and hepatoprotective properties. *P. grandiflorus* has important clinical value because of the active triterpenoid saponins in its roots. However, the biosynthetic pathway of triterpenoid saponins in *P. grandiflorus* remains unclear, and the related genes remain unknown. Therefore, in this study, we assembled a high-quality and integrated telomere-to-telomere *P. grandiflorus* reference genome and combined time-specific transcriptome and metabolome profiling to identify the cytochrome P450s (CYPs) responsible for the hydroxylation processes involved in triterpenoid saponin biosynthesis. Nine chromosomes were assembled without gaps or mismatches, and nine centromeres and 18 telomere regions were identified. This genome eliminated redundant sequences from previous genome versions and incorporated structural variation information. Comparative analysis of the *P. grandiflorus* genome revealed that *P. grandiflorus* underwent a core eudicot γ-WGT event. We screened 211 CYPs and found that tandem and proximal duplications may be crucial for the expansion of CYP families. We outlined the proposed hydroxylation steps, likely catalyzed by the CYP716A/72A/749A families, in platycodin biosynthesis and identified three *PgCYP716A*, seven *PgCYP72A,* and seven *PgCYP749A* genes that showed a positive correlation with platycodin biosynthesis. By establishing a T2T assembly genome, transcriptome, and metabolome resource for *P. grandiflorus*, we provide a foundation for the complete elucidation of the platycodins biosynthetic pathway, which consequently leads to heterologous bioproduction, and serves as a fundamental genetic resource for molecular-assisted breeding and genetic improvement of *P. grandiflorus*.

## Introduction

*Platycodon grandiflorus* (Jacq.) A. DC., commonly known as the balloon flower, is a clump-forming perennial herbaceous species in the Campanulaceae family [1]. It is native to China, Korea, and Japan, and is cultivated in several areas of China. *P. grandiflorus* roots have been used in traditional Chinese medicine for over a millennium, with their use documented in the ‘Shennong Classic of Materia Medica’ [2]. It is commonly used to treat lung, respiratory and intestinal diseases [3–5]. Phytochemical analysis of *P. grandiflorus* has identified several compounds, including triterpenoid saponins, flavonoids, polysaccharides, and phytosterols [6]. Among these, triterpenoid saponins are considered to be the most important active components, exhibiting various pharmacological activities. To date, numerous oleanene-type triterpenoid saponins, such as platycodins, have been isolated from *P. grandiflorus*. These compounds have sugar chains at the C-3 and C-28 positions in the saponin parent nucleus [7].

The increase in available reference genomes has provided a comprehensive spectrum of genes and variations associated with critical traits, such as substance accumulation and environmental adaptation [8–10]. *Platycodon grandiflorus* has three assembled genome versions. The initial draft of the *P. grandiflorus* genome was published by Korean researchers in 2017 [11]. Subsequently, two chromosome-level genomes have been published, one for the Korean bellflower and the other for 4-week-old XJD seedlings (Chinese cultivar) [12, 13]. Owing to technological constraints, previous genome versions have contained several gaps and mismatches. To address these limitations, telomere-to-telomere (T2T) assembly, which utilizes deep sequencing, can be used to assemble high-quality genomes [14].

T2T assemblies of medicinal plants have been reported to provide crucial insights into the identification of genetic elements and regulators of plant development, secondary metabolites, and stress tolerance. For instance, the T2T genome of *Isodon rubescens* f. lushanensis has provided molecular evidence to identify novel genes involved in the diterpenoid biosynthetic pathway and structural variations within diterpenoid synthesis genes [15]. Researchers have also assembled the complete T2T genome of *Scutellaria baicalensis*, systematically analyzed the cytochrome P450 (CYP) family, and characterized the crucial enzymes involved in anthocyanin modification. Previously, we elucidated some genes of the platycodin biosynthetic pathway using transcriptome analysis. However, the complete genome assembly of *P. grandiflorus* is essential for understanding its genetic information.

The platycodin biosynthetic pathway can be summarized into three primary steps. First, the common methylerythritol 4-phosphate (MEP) and mevalonate (MVA) pathways produce the precursors of the terpenoids isopentenyl diphosphate (IPP) and dimethylallyl pyrophosphate (DMAPP), with the MVA pathway being the main contributor to triterpenoid biosynthesis. These precursors then undergo carbon chain extension and cyclization to form the oleanene-type triterpenoid skeleton, β-amyrin. Subsequently, this structure is modified by hydroxylation and glycosylation to form various types of platycodins. Although our previous study confirmed the function of some genes in the intermediate pathway [16], the complete biosynthetic pathway remains unexplored. CYPs comprise the largest family of enzymes that catalyze monooxygenation reactions and are crucial for platycodin biosynthesis and diversification. Therefore, a systematic analysis of CYP would enhance our understanding of platycodin biosynthesis and lay the foundation for the efficient synthesis of active saponins.

In this study, high-throughput chromosome conformation capture (Hi-C), ultra-long oxford nanopore technology (ONT), and PacBio HiFi sequencing strategies were used to assemble the first complete T2T gap-free genome of *P. grandifloru*s. The 593.56 Mb genome was assembled and all sequences were mapped to nine chromosomes. Comparative genomic analyses were performed to gain insights into *the evolution of P. grandiflorus*. The accumulation of major triterpenoid saponins during various growth stages was determined, and the genes involved in the platycodon metabolic pathway were identified using this complete genome. The CYP gene family plays a key role in the diversification of platycodins by catalyzing hydroxylation at various positions in the aglycone nucleus. Systematic identification and characterization of the CYP gene family in *P. grandiflorus* were performed and screened for potential CYPs associated with platycodin biosynthesis. This study provides comprehensive genetic information on *P. grandiflorus* and serves as a basis for understanding the functions of the CYP gene family.

## Results

### Telomere-to-telomere complete genome assembly of *P. grandiflorus*

To achieve a high-quality and complete *P. grandiflorus* genome assembly, the second-generation sequencing (DNBSEQ) and k-mer-based analyses were performed to achieve a high-quality and complete *P. grandiflorus* genome assembly. The results of the depth frequency distribution (K = 19, depth = 51.6) indicated that the estimated genome size of wild *P. grandiflorus* was 557.63 Mb, and the heterozygous ratio was 0.91% (Fig. S1 and Table S1). Based on combined sequencing technologies, ONT ultra-long, HiFi, Hi-C, and next-generation sequencing, 691.3, 34.7, 82.4, and 36.1 Gb data were separately obtained (Tables S2–S6). The sequences were anchored to nine chromosomes, and one gap was found on chromosome 9 (45.67–45.68 Mb). ONT and HiFi reads were mapped to the gap regions to be filled. The final 593.56 Mb T2T genome was generated and aligned to nine chromosomes (Fig. S2 and Table S7). The consistency of the assembled T2T genome was evaluated by mapping Illumina, ONT, and HiFi reads to the genome, which resulted in an approximately 100% mapping rate and coverage (Table S9). Assembly completeness was analyzed using Benchmarking Universal Single-Copy Orthologs (BUSCO), and the complete BUSCO were 97.7% (Table S10). The quality value of the genome assembly was 42.3123, and each chromosome was between 40.70 and 43.27, proving the high accuracy of genome assembly (Table S11).

**Figure 1:**
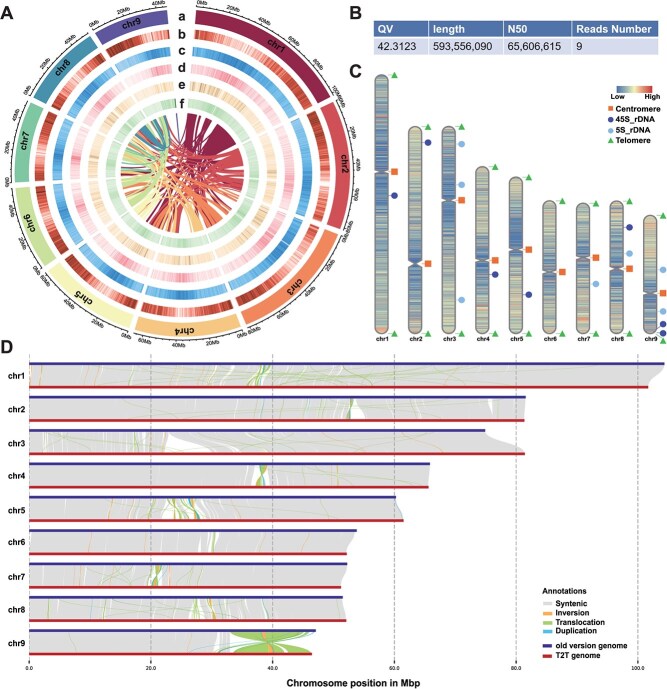
Landscape of *P. grandiflorus* collection and genomic features. (A) Distribution of *P. grandiflorus* genomic features. a-f: chromosomes, gene density, repetitive sequences density, *Copia* density, *Gypsy* density, GC content (window size = 50 kb). (B) Quality value of T2T assembly. (C) Distribution of telomeres and centromeres. The strips of each chromosome represent gene density calculated by bedtools v2.30.3 in 50 kb windows. (D) Structural variations between *P. grandiflorus* T2T genomes and cultivar XJD.

To date, two genome assemblies of the Korean *P. grandiflorus* and the Chinese cultivar XJD have been reported. Compared to previous genome versions, the T2T genome had the longest N50 contig length (approximately 65 Mb) and zero gaps (Table S8), indicating that the T2T genome achieved maximum integrity. T2T assembly addressed the issue of contigs not being anchored to chromosomes, which was present in previous assembly versions. Chromosomal collinearity analysis was performed between T2T and the original genomes (Fig. 1D). A large translocation region on chromosome 9 was rectified by ultra-long reads of the T2T genome to generate the correct structure-variant arrangement. Additionally, the T2T genome eliminated redundant sequences from the previous version of the assembly and corrected the erroneous chromosomal mapping.

### Telomere and centromere characteristics

Repetitive sequences in the telomeric regions were identified, and 18 telomeric regions were predicted on nine chromosomes (Fig. 1B and Table S12). The centromeric region has a special sequence structure: a high tandem repeat density and an extremely low gene density. Based on tandem repeat finder analysis, the density of tandem repeats along with genes was plotted to estimate the centromeric regions (Fig. S3). Tandem repeat monomers with >90% similarity were selected and clustered on each chromosome. The locations of the top nine abundant tandem repeat clusters in each chromosome were drawn, and the most intensive tandem repeat cluster regions were presumed to be centromeric regions (Fig. S4). The longest tandem repeat clusters were subsequently merged to obtain the final centromeric locations (Fig. S5 and Table S13). These telomeric and centromeric regions have not been identified in previous versions of the *P. grandiflorus* genome. As shown in Fig. 1C, little sequence collinearity was observed in the centromeric regions.

### Gene annotation and repeat sequence recognition

A total of 394 829 917 transposons were identified in the genome (66.5%), comprising long terminal repeats (LTR), long interspersed nuclear elements, short interspersed nuclear elements, and DNA transposons, among which LTR retrotransposons (LTR-RTs) constituted 40.72% (Fig. S6 and Table S14). *Gypsy* and *Copia* represent two types of LTR-RTs, and their abundances on nine chromosomes are illustrated in Fig. 1A and Fig. S7. The LTR assembly index score was 25.83, indicating the high-quality of the *P. grandiflorus* T2T assembly (Fig. S8). All predicted tandem repeat sequences were counted according to their length. Microsatellites (1–9 bp) comprised the majority of 683 710 tandem repeat sequences (Table S15). By combining ab initio, homology (four species: *Arabidopsis thaliana*, *Codonopsis lanceolata*, *Helianthus annuus*, and *Panax ginseng*), and RNA-seq approaches, we identified 27 808 protein-coding genes in the *P. grandiflorus* T2T genome and assessed gene annotation using BUSCO (Tables S1 and S17). The average coding sequence length per gene was 1115 bp, and its distribution is shown in Fig. S9. A total of 25 245 (90.78%) predicted genes were annotated to seven functional databases, of which 24 592 were annotated in the Interpro database and 9658 genes were homologous to proteins in the Kyoto Encyclopedia of Genes and Genomes (KEGG) pathways (Fig. S10 and Table S18). In total, 159 and 952 were annotated as noncoding RNAs (miRNAs and tRNAs), respectively. Various types of rRNAs (3021) and snRNAs (1112) were identified, accounting for 0.28% and 0.04% of the T2T genome, respectively (Table S19).

### Genome evolution

Orthologous clustering was performed for 13 species, and their protein sequences were clustered into 90 066 gene families. A total of 8107 gene families were identified in 13 species, and 3154 gene families were specific to the *P. grandiflorus* genome (Table S20). Gene ontology (GO) and KEGG annotations were performed for specific gene families comprising 4138 unique paralogs. The results indicated that the most enriched GO terms were apoplast, ATP hydrolysis activity, ADP binding, and glycosyltransferase activity, and the most significant KEGG enrichment pathway was the plant–pathogen interaction (Fig. S11). Shared gene families in *P. grandiflorus*, *C. lanceolata*, *H. annuus*, and *A. thaliana* were analyzed, and functional enrichment was performed on 11 046 shared gene families in the four species and 3493 unique gene families in *P. grandiflorus* (Fig. S12). The functions of the mutual gene families in the four species were predominantly associated with the translation process (Fig. S13).

**Figure 2:**
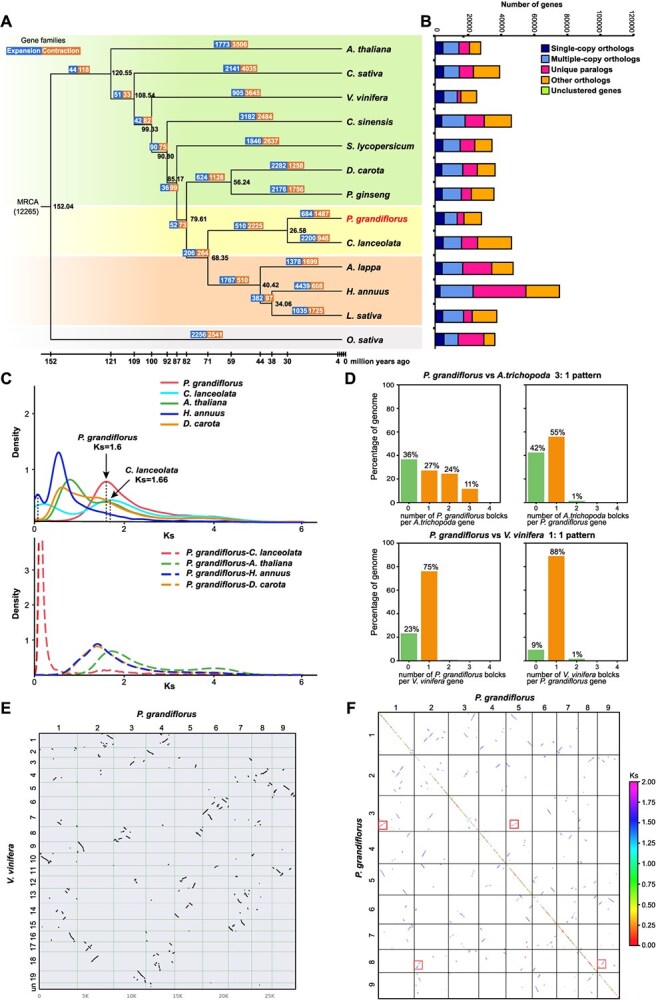
Evolution of the *P. grandiflorus* T2T genome. (A) Phylogenetic tree comprises 13 species. (B) Number of homologous genes identified in 13 species. (C) Density plot of synonymous substitution rate (Ks) distribution in *P. grandiflorus*, *C. lanceolata*, *A. thaliana*, *H. annuus*, *D. carota*. (D) The syntenic depth ratios between different species genome pairs. (E) Dot plots of orthologues between *P. grandiflorus* and grape, suggesting a 1:1 syntenic relationship. (F) Ks dot plot of *P. grandiflorus* genome. The color of the dot indicates the Ks of the gene pair. Four boxes indicate the colinear gene regions.

Single-copy orthologous genes detected by orthologous clustering were used for subsequent evolutionary analyses (Fig. 2B and Table S21). We constructed a phylogenetic tree based on 508 single-copy orthologs identified in *P. grandilflorus* and 12 other species to estimate their divergence times (Fig. 2A). *Oryza sativa* was selected as the outgroup. The rooted tree showed that *P. grandiflorus* diverged from the same plant family *C. lanceolata* 26.58 million years ago (Mya). Campanulaceae and Asteraceae diverged approximately 68.35 Mya. Furthermore, we analyzed the expanded and constructed gene families in all species of the phylogenetic tree. A total of 12 265 gene families were detected in the most recent common ancestors. In the *P. grandiflorus* genome, 684 gene families (2405 genes) and 1487 gene families (2320 genes) were contracted. The significant expanded genes were mainly related to transcriptional regulation, whereas the significant contracted genes were primarily involved in oxidoreductase activity (Fig. S14). Sixteen genes involved in diterpenoid, sesquiterpene, and triterpenoid biosynthesis were identified in *P. grandiflorus* (Table S22).

Genomic collinearity analysis of *P. grandiflorus* identified 198 intragenomic blocks, including 4168 collinear genes. We used the age distribution of the duplicated genes to identify significant peaks in gene duplications consistent with the whole-genome duplications (WGDs). A peak around Ks 1.6, representing the WGD event of *P. grandiflorus*, was identified (Fig. 2C). A peak of duplication with a median Ks around 1.66 was also found in *C. lanceolata*, and the duplication event occurred after the divergence of *P. grandiflorus* and *C. lanceolata* (Ks 1.7). To confirm that *P. grandiflorus* underwent ancient WGD, we compared the syntenic depth ratios of the *P. grandiflorus*, *Amborella trichopoda,* and *Vitis vinifera* genomes. We observed an overall one-to-one syntenic depth ratio between *P. grandiflorus* and *Vitis vinifera* (Fig. 2D), and confirmed the existence of the core eudicot triplication (γ-WGT) in the two species. The syntenic depth ratio of 1:3 between the *P. grandiflorus* and *Amborella trichopoda* genomes further supports this inference. In the dot plot of the *P. grandiflorus* genome, each chromosome contained two matched regions (Fig. 2F). These results suggest that *P. grandiflorus* experienced an ancient γ-WGT event.

**Figure 3:**
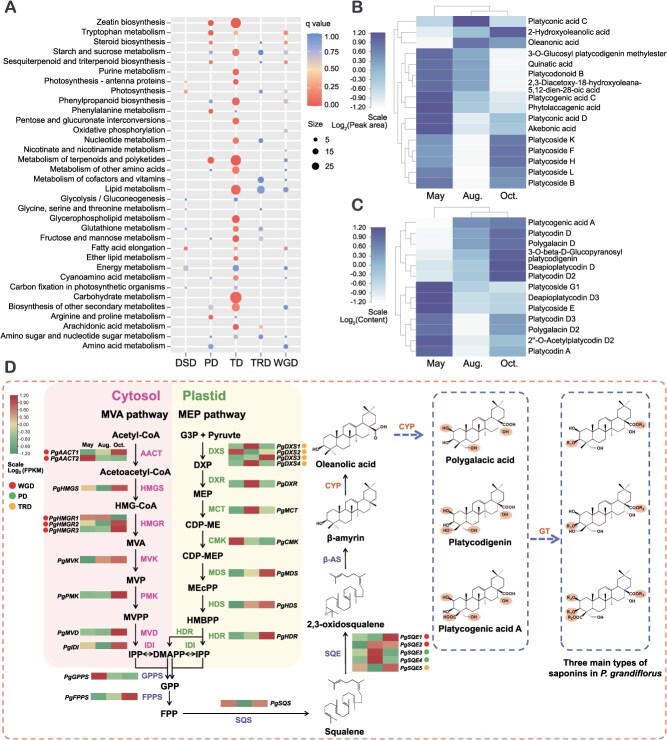
Gene duplication and triterpenoid biosynthesis in *P. grandiflorus*. (A) KEGG enrichment analysis of duplicated genes belonging to expanded gene families. (B) Relative contents of triterpenoid and saponins in roots of *P. grandiflorus* at different stages. The data were obtained from metabonomics. (C) Thirteen saponins content in roots of *P. grandiflorus* at different stages. These compounds are quantified by UPLC-Orbitrap-MS/MS. (D). Triterpenoid saponins biosynthetic pathways and related genes expression profiles. The expression patterns of related genes were displayed by heatmap, and colored circles represent gene duplicated types. AACT: acetyl-CoA C-acetyltransferase; HMGS: hydroxymethylglutaryl-CoA synthase; HMGR: hydroxymethylglutaryl-CoA reductase; MVK: mevalonate kinase; PMK: phosphomevalonate kinase; MVD: diphosphomevalonate decarboxylase; DXS: 1-deoxy-D-xylulose-5-phosphate synthase; DXR: 1-deoxy-d-xylulose-5-phosphate reductoisomerase; MCT: 2-C-methyl-d-erythritol 4-phosphate cytidylyltransferase; CMK: 4-diphosphocytidyl-2-C-methyl-d-erythritol kinase; MDS: 2-C-methyl-d-erythritol 2,4-cyclodiphosphate synthase; HDS: (E)-4-hydroxy-3-methylbut-2-enyl-diphosphate synthase; HDR: 4-hydroxy-3-methylbut-2-en-1-yl diphosphate reductase; IDI: isopentenyl-diphosphate Delta-isomerase; GPPS: geranyl diphosphate synthase; FPPS: farnesyl diphosphate synthase; SQS: squalene synthase; SQE: squalene monooxygenase; β-AS: β-amyrin synthase; CYP450: cytochrome P450; GT: glycosyltransferase.

The evolution of species is accompanied by gene replication. Using DupGen_finder, we identified 16 991 duplicated genes in the *P. grandiflorus* genome that were induced by WGD, tandem duplication (TD), proximal duplication (PD), transposed duplication (TRD), and dispersed duplication (DSD) (Table S23) [17]. Duplicate genes with expansion families were selected for KEGG enrichment analysis. The abundance of duplicates in the biosynthesis of other secondary metabolites was primarily because of TD events (Fig. 3A). Notably, five expansion genes annotated in the sesquiterpenoid and triterpenoid biosynthesis pathways were named SQE-encoding genes (*SQE1*-*SQE5*). Non-synonymous substitutions (Ka) of single-copy genes were calculated, and 52 genes were identified as significantly positive, with a Ks/Ka ratio > 1 (FDR < 0.05). The most significantly positively selected genes were enriched for intracellular photosynthesis, transcription, and metabolism (Fig. S15).

### Metabolomics analysis of *P. grandiflorus* roots collected in different growing stages

Previously, we collected 2-year-old *P. grandiflorus* from January to December to investigate the total saponin content. The results demonstrated that saponin content in the roots decreased from January to February, increased until May, and decreased to its lowest level in August. The total saponin content continuously increases to its highest level until October and then declines again. According to the trend of total saponin content, we collected fresh *P. grandiflorus* roots in May, August, and October and subsequently frozen them at −80°C for further metabolomic and transcriptome analyses.

To further investigate the dynamic accumulation of triterpenoid saponins in *P. grandiflorus*, we performed a widely targeted metabolomic analysis. The peak areas of all metabolites represent their relative contents in the different samples. Principal component analysis (PCA) showed that two principal components, PC1 and PC2, contributed 28.22% and 15.13%, respectively, to the differences in secondary metabolites of different growing stages of *P. grandiflorus* (Fig. S16). The metabolic profiles of the May samples were significantly different from those of the August and October samples, respectively. The accumulation patterns of metabolites in each sample were shown in Fig. S17. Consistent with the PCA results, the heatmap showed similar metabolite patterns among the three groups. A total of 1, 350 metabolites, including terpenoids (12.74%), phenolic acids (11.41%), flavonoids (7.11%), and others, were obtained from metabolomics, among which triterpenoid components were the focus of this study (Fig. S18). After data processing, 16 pentacyclic triterpenoids and oleanane-type saponins were identified (Fig. 3B). The content of *P. grandiflorus* roots at different stages was measured based on their peak areas, with most showing higher levels in May and October. Considering the limitations of metabolomic analyses, we additionally determined 13 saponin contents via UPLC-Orbitrap-MS/MS, and the highest saponin content was observed in May and October (Fig. 3C).

### Mining of genes involved in triterpene saponins biosynthesis in *P. grandiflorus*

Platycodins, oleanane-type triterpenoid saponins, are pharmacodynamic compounds found in *P. grandiflorus*. Platycodins are derived from the common terpenoid precursors IPP and DMAPP, which are produced via the MVA pathway in the cytoplasm and the MEP pathway in the plastid. IPP is synthesized via a six-step condensation process in the MVA pathway, which is a seven-step catalytic reaction in the MEP pathway. The MVA pathway contributes more significantly to the biosynthesis of triterpenoid saponins [18]. IPP and DMAPP are interconverted by isopentenyl diphosphate isomerase. IPP and DMAPP are further condensed into geranyl pyrophosphate and then into farnesyl pyrophosphate (FPP). The two FPPs continue to condense to form squalene under catalysis by squalene synthase. Squalene is epoxidized to 2,3-oxidosqualene by squalene epoxidase (SQE), and then cyclized to generate the oleanane-type pentacyclic triterpenoid skeleton β-amyrin. β-amyrin undergoes various modifications, such as oxidation, hydroxylation and glycosylation under cytochrome P450 monooxygenases (CYPs) and UDP-glycosyltransferase (UGT) to produce various platycodins (Fig. 3C).

Based on the *P. grandiflorus* T2T genome, we applied KEGG annotation and a homologous protein search to identify genes coding for catalytic enzymes involved in triterpenoid biosynthesis. The number of full-length genes related to triterpenoid biosynthesis is shown in Fig. 3C, of which the majority were single-copy genes. The multicopy of genes, two *PgAACTs* and three *PgHMGRs* in the MVA pathway, were caused by WGD events. TD contributed to multiple copies of the four *PgDXSs* in the MEP pathway. Five *PgSQEs* were duplicated because of WGD, TD, and PD events.

To further characterize the identified genes, the transcriptomes of *P. grandiflorus* roots were analyzed at different growth stages (May, August, and October) in three biological replicates. A total of 57.97 Gb of clean data were obtained, with the Q30 of all samples above 90%. The clean reads were mapped to the T2T genome, and the mapping ratios ranged from 92.04% to 95.24% (Table S24). The expression of identified genes related to 2,3-oxidosqualene biosynthesis showed that 19 genes (*PgAACTs*, *PgHMGS*, *PgHMGR2/3*, *PgMVK*, *PgPMK,* and *PgMVD* in the MVA pathway; *PgDXS2/3*, *PgCMK*, *PgMDS*, *PgHDS*, and *PgHDR* in the MEP pathway; *PgIDI*, *PgGPPS*, *PgFPPS*, *PgSQS*, and *PgSQE1*/5) in the midstream pathway were expressed at higher levels in May and October. Genes derived from gene duplications showed varied expression patterns among the different growth stages.

### Characterization analysis of OSC genes in *P. grandiflorus* genome

OSCs are the rate-limiting enzymes of triterpene/sterol biosynthesis, generating diverse scaffolds from 2,3-oxidosqualene in a very complex multistep catalytic process involving substrate binding and folding, epoxide protonation, sequential cyclization or rearrangement, and reaction termination through deprotonation or water capture [19]. We performed a BLAST homology search against the *P. grandiflorus* T2T genome using 162 known OSC proteins from other species as queries (Table S25). Thirteen OSC candidate genes were screened (*PgOSC1–13*), with amino acid (aa) sequence lengths ranging from 583 to 776 aa. Thirteen PgOSC proteins contained three conserved domains: squalene/hopene cyclase N- and C-terminal domains (PF13249 and PF13243, respectively), prenyltransferase, and squalene oxidase repeats (PF00432) (Fig. S19A). The N-terminal domain PF13249 was absent in PgOSC1 and incomplete in PgOSC8, whereas the C-terminal domain PF13243 was incomplete in PgOSC4. Sequence alignment and conserved motif prediction analyses were performed on 13 PgOSC proteins, which displayed one conserved catalytic Asp residue-containing ‘DCTAE’ motif involved in substrate binding, four ‘QW’ motifs (except for three ‘QW’ motifs in PgOSC4, and two ‘QW’ motifs in PgOSC8), and OSC-specific ‘MWCYCR’ motif (except for the mutation in PgOSC5/9/11/12/13) (Figs S19B and S20). The ‘QW’ motif is contribute to OSC structure stability, which help stabilize carbocationic intermediates during cyclization process. Incomplete ‘QW’ motif may affect OSC activity, which provide a valuable reference to further elucidate the functional roles of putative *PgOSC* genes.

**Figure 4:**
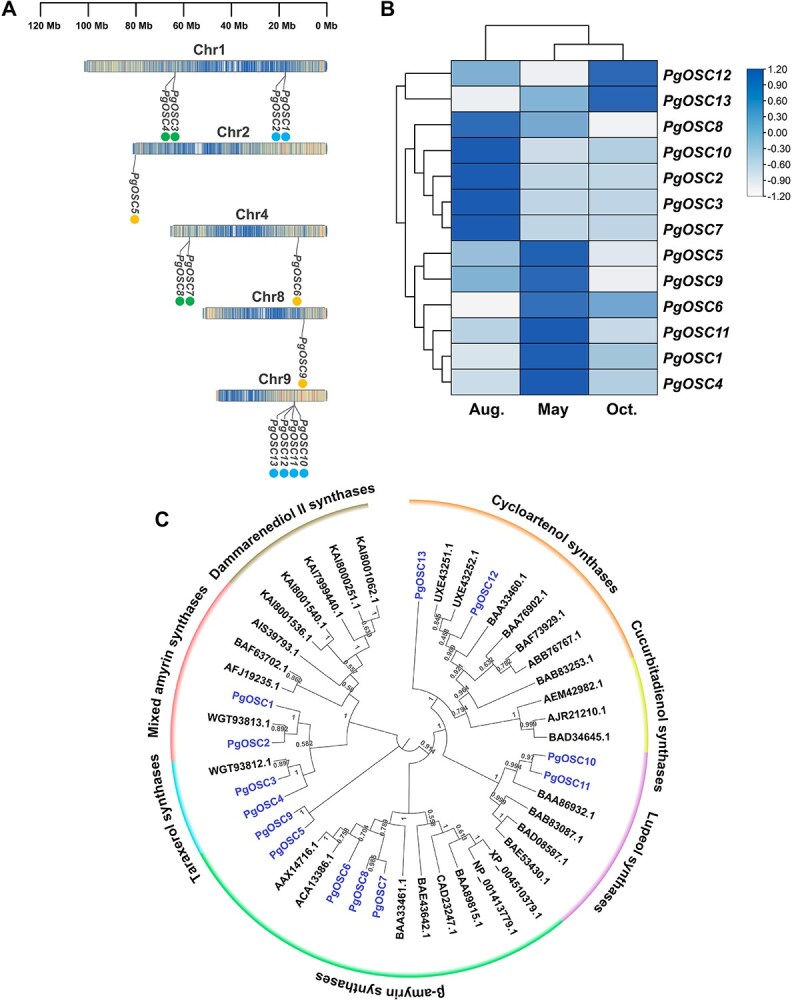
Chromosome location, gene expression patterns and phylogenetic analysis of *PgOSCs* genes identified in *P. grandiflorus* T2T genome. (A) Chromosome location of 13 candidate *PgOSCs*. Only chromosomes 1, 2, 4, 8, and 9 containing the *PgOSCs* are shown. Colored circle patterns represent tandem, proximal and transposed duplicates, respectively. (B) Expression patterns of PgOSCs in *P. grandiflorus* roots at different growing stages. (C) A maximum-likelihood tree of PgOSCs proteins with characterized OSCs in other species. The specific information of other plant OSCs was listed in Table S25.

To infer the evolution of PgOSCs, a maximum-likelihood phylogenetic tree was constructed using 13 PgOSC proteins and OSCs characterized in other species (Table S26). According to phylogenetic relationships, all PgOSCs were distributed among the five branches (Fig. 4C). PgOSC1, PgOSC2, PgOSC3, and PgOSC4 clustered in one branch. PgOSC1 and PgOSC2 were closely grouped with the functionally characterized mixed amyrin synthase of *C. lanceolata* (WGT93813.1), whereas PgOSC3 and PgOSC4 clustered with *C. lanceolata* taraxerol synthase (WGT93812.1). PgOSC6, PgOSC7, and PgOSC8 were categorized as β-amyrin synthase (β-AS). PgOSC10 and PgOSC11 were located in the lupeol synthase cluster, whereas PgOSC12 and PgOSC13 were grouped together with cycloartenol synthase. Specifically, PgOSC5 and PgOSC9 clustered in a single branch. For further characterization, we constructed a tree using PgOSC5, PgOSC9 and other 162 OSC proteins, and found that two proteins were grouped with *Gossypium arboreum* β-AS (Fig. S21 and Table S25). Thus, five *PgOSCs* (*PgOSC5—PgOSC9*) were categorized as β-AS genes. Seven genes (*PgOSC1*, *PgOSC2*, and *PgOSC5—PgOSC9*) were probably involved in β-amyrin biosynthesis.

Thirteen *PgOSC* genes were identified on chromosomes 2, 4, 6, 8, and 9. Chromosomes 2 and 8 contained only one *PgOSC* gene, whereas chromosomes 1, 4, and 9 harbored multiple *PgOSC* genes (Fig. 4A). *PgOSC1* and *PgOSC2* (putative mixed amyrin synthase) and *PgOSC3* and *PgOSC4* (putative taraxerol synthase) were found adjacent to chromosome 1 and were identified as tandem duplicated and proximal duplicated gene pairs, respectively. *PgOSC7* and *PgOSC8* (putative β-AS), with close locations on chromosome 4, were proximal repeat gene pairs. Additionally, four *PgOSCs* congregated on a region of chromosome 9, with *PgOSC10-PgOSC11* (putative lupeol synthase), and *PgOSC12-PgOSC13* (putative cycloartenol synthase) were two tandem repeat pairs.

The expression of the 13 *PgOSCs* in *P. grandiflorus* roots at different growth stages was analyzed using transcriptome datasets (Figure 4B-D). Most *PgOSCs* were expressed at high levels in the roots in May and August, except for *PgOSC12* and *PgOSC13,* which were expressed at high levels in October. The proximal repeats (*PgOSC7* and *PgOSC8*), transposed repeats (*PgOSC5*, *PgOSC6*, and *PgOSC9*), and tandem repeats (*PgOSC12* and *PgOSC13*) exhibited similar expression patterns.

### *P. grandiflorus* T2T genome contains 211 CYP genes

**Figure 5:**
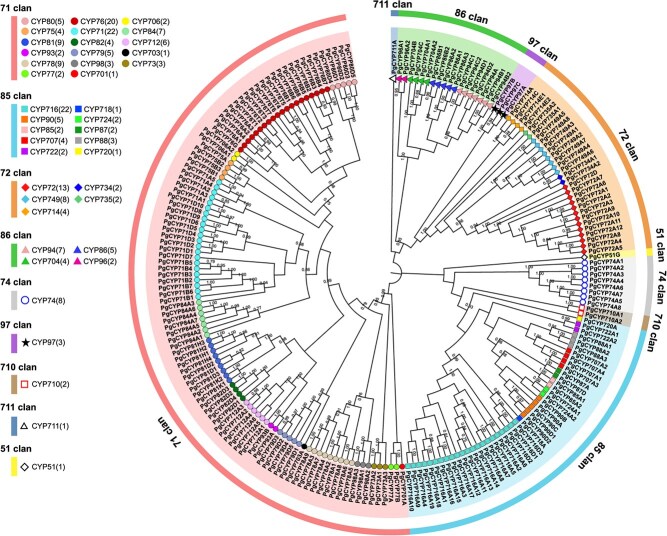
Phylogenetic analysis of 211 CYPs in *P. grandiflorus*. Clans and families are separately indicated using color strips and symbols.

For a more comprehensive analysis of the CYP gene family, 211 sequences were screened by combining protein annotation, Hidden Markov Model (HMM) search (PF00067), and homologous sequence alignment. A total of 211 non-redundant proteins (> 300 aa) were used for further analysis. The families and clans of the CYPs were identified using a National Center for Biotechnology Information (NCBI) BLAST search (https://blast.ncbi.nlm.nih.gov/Blast.cgi), and their phylogenetic relationships with CYPs in other species were determined. After confirming the classification of the 211 CYPs, they were named based on their subfamilies and chromosomal localization. A neighbor-joining phylogenetic tree of 211 CYP was constructed, and CYPs belonging to the same subfamilies were clustered in one branch, indicating the reliability of the classification of 211 CYPs (Fig. 5). Clans are higher-order groupings of CYP families, and nine clans (41 subfamilies) have been identified in *P. grandiflorus*. Clan 71 belonged to the A-type CYP, containing 105 CYPs, whereas the other eight clans belonged to the non-A-type. As shown in the phylogenetic tree, 211 CYPs were clustered into three major branches. Clan 71 constituted a single branch; clans 72, 86, 97, and 711 grouped into one cluster; and clans 74, 85, 710, and 51 formed another cluster. Clans 71, 85, 72, and 86 were multifamily clans, whereas clans 74, 97, 710, 711, and 51 were single-family clans.

### CYPs in *P. grandiflorus* exhibit prevalent gene duplication events

A total of 211 CYPs genes were unevenly distributed across the nine chromosomes, with 114 of the 211 genes located on chromosomes 1, 2, and 3 (Fig. S22). Notably, genes with adjacent locations likely belonged to the same subfamily, which may have been caused by gene duplication. According to the duplicated gene analysis results, 177 CYPs were duplicated because of WGD, TD, PD, TRD, or DSD events (Fig. S23 and Table S27). Among 177 CYPs, 63 were tandem duplicated genes, indicating that TD was the main driving force for the expansion of the CYP gene family in *P. grandiflorus*. This pervasive phenomenon of gene duplication has resulted in the emergence of diverse and intricate CYP450 families across species.

Among the 211 CYPs in *P. grandiflorus*, eight CYPs belonged to the CYP78A subfamily and one belonged to the CYP735A subfamily, whereas 39 CYPs mainly belonged to CYP71A/B/D and CYP76B/T (Table S28). CYP78A members have been reported to regulate seed and fruit size in multiple species [20]. Expansion within the CYP71D subfamily has been reported to drive the heterocyclization of tanshinone synthesis in *Salvia miltiorrhiza* [21]. The contraction and expansion of these CYPs may affect the secondary metabolite diversity and plant physiological functions.

### Conserved motifs and gene structures of CYPs in *P. grandiflorus*

Fifteen motifs were identified in the 211 CYP proteins. Clan 71 contained 14 motifs (all except motif 12), whereas the other non-A-type clans contained fewer motifs (Fig. S24). CYPs within the same clan showed similar motif compositions (Fig. S25). Six conserved motifs are characteristic of CYP proteins: proline-rich motif (motif 8), C-helix motif (motif 6), I-helix motif (motif 4), K-helix motif (motif 2), PERF motif (motif 3), and heme-binding motif (motif 1). The proline-rich motif ‘PPGPx(P/G)xP’ is present following the N-terminal signal-anchor-sequence and a short hydrophilic linker sequence, and is considered crucial for the correct orientation of CYP enzymes to the membrane [22]. Tryptophan and arginine in the C-helix motif ‘WxxxR’ interact with the propionate side chain of heme. The K-helix motif ‘ExxR’ and PERF motif form the E-R-R (glutamate-arginine-arginine) triad, which is linked to heme-binding [23]. The I-helix motif ‘(A/G)Gx(D/E)T(T/S)’ has been shown to be related to proton delivery [24]. Notably, the heme-binding motif ‘FxxGxRxCxG’ is reported to be the most crucial of these conserved motifs because it facilitates association with the heme cofactor in the active site [25].

We used TBtools software to visualize the structures of 211 CYPs and found that CYPs in the same subfamily may possess a similar composition of coding sequences and untranslated regions (Fig. S26). The number of exons and introns in the 211 CYPs varied from 1 to 16 and 0 to 15, respectively (Fig. S27). Most A-type CYPs (clan 71) possessed two exons and one intron. *PgCYP97A* contained the largest number of exons and introns.

### *Cis*-acting elements of CYPs

The upstream 2000 bp of each CYPs was extracted for *cis*-acting element prediction, and the results showed no explicit regular relationships of CYPs in the same subfamily. Vital elements can generally be classified into four categories: developmental (such as CAT-box, GCN4_motif, and O_2_-site), light (such as G-box, Box 4, and GT1-motif), hormone (such as TGA-element, ABRE, and TGACG-motif), and stress (such as ARE, MBS, and LTR) responsiveness (Fig. S28). Thirty types of light-responsive elements were predicted in 211 CYPs, with Box 4 and G-box accounting for the largest proportion. Four stress-responsive elements, namely ARE, MBS, LTR, and TC-rich repeats, were predicted. Eleven hormone-responsive elements were predicted, of which ABRE responding to abscisic acid had the highest frequency. Four types of elements related to plant development were found in CYPs: CAT-box, GCN4_motif, O_2_-site, and circadian, which are involved in the circadian control and expression of zein metabolism, endosperm, and meristem.

### Candidate CYPs related to the diversity of triterpenoid saponins in *P. grandiflorus*

Previously, researchers have shown that the *CYP716A140v2* gene in *P. grandiflorus* catalyzes β-amyrin to produce oleanolic acid [26]. Oleanolic acid is the pentacyclic triterpene skeleton of *P. grandiflorus*, which is generated by oxidation at C28 of β-amyrin. Based on their structural characteristics, the oleanane-type saponins in *P. grandiflorus* are mainly divided into three types: platycodigenin, platycogenic acid, and polygalacic acid [27]. Three triterpenes, platycodigenin, platycogenic acid A, and polygalacic acid, are the precursors of these three types of saponins.

Members of the CYP51/71/72/85/ clans have been identified to be involved in triterpenoid biosynthesis [28]. Previous studies on the CYP716A family in some species, including *Glycyrrhiza uralensis*, soapbark tree, and *Aralia elata*, have shown that *CYP716A* genes catalyzes oxidation at C-28 and hydroxylation of C-2α, C-6β, C-16α, and C-22α [29–32]. *MtCYP72A* in *Medicago truncatula* has been reported to be responsible for C-2β and C-23 hydroxylation [33]. *CpCYP749A63* in *Crataegus pinnatifida* catalyzes C-24 hydroxylation of oleanolic acid [34]. The CYP93E family also acts as a C-24 hydroxylase; however, it is unique to leguminous species.

**Figure 6:**
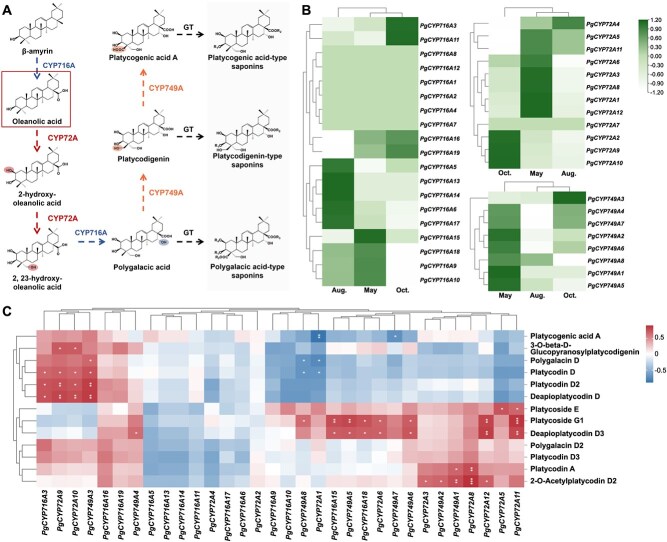
The down-stream biosynthetic pathway for oleanane type triterpenoid saponins in *P. grandiflorus*. (A) The proposed biosynthetic pathway of platycodins. The positions of modification and the enzymes involved are shown in the same color. (B) The expression pattern of related genes. (C) The correlations between CYPs expression and saponins content. *p < 0.05; **p < 0.01.

Based on these findings, we focused on the CYP716A, CYP72A, and CYP749A subfamilies and investigated the downstream biosynthetic pathways of platycodins in *P. grandiflorus* (Fig. 6A). We hypothesized that genes belonging to the CYP716A, CYP72A, and CYP749A subfamilies participate in triterpenoid biosynthesis in *P. grandiflorus*. Specifically, the CYP72A family of enzymes catalyzes the hydroxylation of oleanolic acid at the C2 and C23 positions. The CYP716A family catalyzes C-16 hydroxylation to produce polygalacic acid, which is then converted to platycodigenin and platycogenic acid A under the C-24 hydroxylation and oxidation activities of CYP749A.

A total of 19 CYP716A, 12 CYP72A, and 8 CYP749A genes were screened from the T2T genome. Their expression patterns in the roots collected at different months were shown in Fig. 6B. Among the CYP716A genes, four CYPs (*PgCYP716A9*/*10*/*15*/*18*) were expressed at high levels in May, and four CYPs (*PgCYP716A3*/*11*/*16*/*19*) were expressed at high levels in October. All CYP72A genes showed the highest expression levels in May and October, except for *PgCYP72A4* and *PgCYP72A7*. Eight CYP749A genes were highly expressed in May and October. Since the content of most platycodins was higher in May and October, therefore CYPs showing correspondingly high expression levels might play important roles in platycodin biosynthesis.

To further analyze the correlation between CYPs expression and platycodin accumulation, the Pearson correlation coefficient was calculated between the FPKM values of CYP716A, CYP72A, and CYP749A and the platycodin content (Fig. 6C). *PgCYP716A3* was significantly and positively correlated with platycodin D, platycodin D_2_, and deapioplatycodin D (*p* < 0.05). *PgCYP716A15* and *PgCYP716A18* were positively correlated with platycoside G1 and deapioplatycodin D_3_. Seven CYP72A genes (*PgCYP72A3*/*5*/*8*/*9*/*10*/*11*/*12*) significantly positively correlated with the content of at least one triterpenoid saponin. *PgCYP749A7* expression was negatively correlated with platycogenic acid A (*p* < 0.05), whereas the other seven CYP749A genes were positively correlated with at least one platycodin gene (*p* < 0.05). Additionally, we downloaded the protein sequences of functionally characterized CYPs that mediate hydroxylation at different positions and used them to construct a maximum-likelihood tree with 211 CYPs identified from the T2T genome. *PgCYP716A18* and *PgCYP716A19* were grouped with other C28 oxidases belonging to the CYP716A subfamily (Fig. S29). *PgCYP716A15* and *PgCYP716A16* clustered with *AeCYP716A355* in *A. elata*, which was characterized as a C16β-hydroxylase. Three CYP749A genes (*PgCYP749A3*, *PgCYP749A5*, and *PgCYP749A8*) were grouped with the C24-hydroxylase *CpCYP749A63* gene identified in *C. pinnatifida*.

## Discussion

As a vital member of the Campanulaceae family, *P. grandiflorus* is known to contain significant medical value. Currently, the T2T gap-free genome assembly strategy has been applied to humans [35], prokaryotes [36], crops [37], and medicinal plants. The T2T genomes of *Panax ginseng* [35], *Gynostemma pentaphyllum* [38], and *S. baicalensis* Georgi [39] have been reported to facilitate genetic information probing. Given the quality of the genome assembly and the different evolutionary patterns of Korean and Chinese *P. grandiflorus*, we assembled the T2T genome of wild *P. grandiflorus* collected from Tongcheng City, Anhui Province, China.

We assembled a 593.56 Mb T2T genome of wild *P. grandiflorus*, including nine centromere and 18 telomere regions. This T2T assembly achieved gapless and zero mismatches for nine chromosomes and was the first to predict centromeres and telomeres. Centromeres are unique chromatin structures that drive chromosomal segregation, and contain numerous tandem repeats. The most intense tandem repeat clusters were distributed in the nine centromeres of *P. grandiflorus*. The quality value of the T2T genome was 42.3123 and that of complete BUSCO was 97.7%. Compared with the previous assembly version (GWHARYT00000000.1, https://ngdc.cncb.ac.cn/gwh), the T2T genome eliminates redundant sequences and improper chromosome localization, and ultra-long reads have elucidated the structural variations of chromosomes.

Based on the evolutionary analysis, we assessed the phylogenetic relationship between *P. grandiflorus* and other species. In the Campanulaceae family, species differentiation of *P. grandiflorus* and *C. lanceolata* (*Codonopsis* genus) probably occurred at 26.58 Mya. By constructing an ancestral model for the branches of *P. grandiflorus* and *C. lanceolata*, we predicted that 2405 genes (684 gene families) and 2320 genes (1487 gene families) would contract. Among these genes, 16 were annotated as being involved in terpene biosynthesis. The genome size was smaller in *P. grandiflorus* than that of *C. lanceolata* (genome size: 1273 Mb) and *Adenophora tetraphylla* (genome size: 2.48 Gb) of the Campanulaceae family [40,41]. The large variation in the genome size of different plants is derived primarily from the amplification of retrotransposons and WGD events [42,43]. Similar to *V. vinifera*, the *P. grandiflorus* genome underwent a γ-WGT event associated with the early diversification of core eudicots. The 1:1 syntenic relationship supported the γ-WGT event shared between *P. grandiflorus* and *V. vinifera*. The 3:1 syntenic blocks between *P. grandiflorus* and *A. trichopoda* also confirmed the occurrence of γ-WGT.

Platycodins are well known to be the main components of *P. grandiflorus* and have shown a variety of pharmacological activities in clinical practice. Metabolomic and transcriptome analyses were performed at different growth stages of *P. grandiflorus* to evaluate platycodins accumulation patterns. Approximately 170 terpenes (12.74%) were identified, 16 of which were pentacyclic triterpenoids and oleanane-type saponins. Thirteen platycodins were also quantitatively analyzed, and these saponins mainly accumulated in May and October. The biosynthesis of various platycodins depends on the catalytic steps related to CYP and UGT. Based on the gap-free genome assembly, we identified coding genes involved in the platycodins synthetic pathway, including 13 OSCs and 211 non-redundant CYPs. Gene duplication is the primary reason for multicopy gene formation, and five gene duplication types have been predicted (WGD, TRD, TD, DSD, and PD) [44]. Notably, WGD and TRD increased the copy numbers of the MVA and MEP pathway genes. Thirteen OSCs and 211 CYP450s mainly underwent TD and PD, respectively. The duplication types of TD and PD may have played a crucial role in the evolution of platycodin biosynthesis. Among the 376 *MsCYPs* in *Medicago sativa*, 112 were involved in TD. TD events have been reported to be a major driver of the expansion of the CYP family in *P. grandiflorus* and *M. sativa* [45].

Hydroxylation and glycosylation are the final catalytic steps for platycodin formation. The CYP gene family is responsible for the C28-carboxylation of oleanolic acid and its subsequent hydroxylation. A total of 211 CYPs were classified into nine clans, including 105 A-type CYPs (clan 71) and 106 non-A-type CYPs (the remaining eight clans). The subfamily composition of the CYP gene family in *P. grandiflorus* was very similar to that identified in *Aralia elata* [46].

The CYP716A gene family has been shown to have conserved functions; it catalyzes a sequential three-step oxidation at the C-28 position of β-amyrin [28,47].CYP716A catalyzes the formation of oleanolic acid in *P. grandiflorus*; however, the underlying pathway remains unclear. Three main types of platycodins have been isolated from *P. grandiflorus,* and their precursor structures (platycodingenin, platycogenic acid A, and polygalacic acid) have been identified. To predict the biosynthetic pathway from oleanolic acid to the three types of precursors, we drew and hypothesized the order of hydroxylation at different positions of oleanolic acid (Fig. 6A). Based on the assumed pathway and information regarding the CYP gene family that catalyzes hydroxylation at different sites of pentacyclic triterpenoids in other species, candidate CYPs were screened for each catalytic step [48]. The CYP72A gene family is speculated to catalyze the C2- and C23-OH of oleanolic acid, whereas the CYP716A gene family may catalyze C16-OH to form polygalacic acid. Furthermore, the CYP749A gene family catalyzes the C24-OH and C24-COOH modifications of polygalacic acid, converting it to platycodigenin and platycogenic acid A, respectively. Among the 211 CYPs, 19 belonged to the CYP716A gene family, 12 to the CYP72A gene family, and 8 to the CYP749A gene family. Consistent with the pattern of platycodin accumulation, eight CYPs of the CYP716A family, 10 of CYP72A, and eight of CYP749A were expressed at higher levels in *P. grandiflorus* collected in May and October (Fig. 6B). To further identify the potential CYPs that participate in platycodin biosynthesis, we performed a correlation analysis between gene expression and platycodin content (Fig. 6C). Based on the correlation relationships and time-specific expression patterns, three *PgCYP716As* (*PgCYP716A3*/*15/18*), eight *PgCYP72As* (*PgCYP72A3/5/6/8/9/10/11/12*), and seven *PgCYP749As* (*PgCYP749A1/2/3/4/5/6/8*) were speculated to play crucial roles in playcodins biosynthesis. These results showed a significant positive correlation with at least one saponin component. However, the functions of these genes require further investigation in future studies.

In this study, we preliminarily identified candidate CYP genes using bioinformatic analyses. However, their catalytic functions require experimental validation, including enzyme assays, gene knockout studies, and overexpression studies. This study provides a foundation for further research and comprehensive elucidation of the biosynthetic pathway of platycodins, consequently leading to heterologous bioproduction and providing a fundamental genetic resource for molecular-assisted breeding and genetic improvement of *P. grandiflorus*.

## Conclusion

Herein, we report the first T2T reference genome of *P. grandiflorus*, along with the metabolomics and transcriptomics of *P. grandiflorus* roots collected at different growth stages. A combination of comparative genomic approaches has been used to elucidate the evolution of *P. grandiflorus*. Additionally, we explored the biosynthesis of natural platycodins and analyzed the CYP450 gene family based on a gap-free genome. We hypothesized the synthetic pathways in which the CYP450 gene family is involved and analyzed candidate CYPs. Therefore, this study provides new insights into the hydroxylation of saponin precursors in *P. grandiflorus* and serves as a basis for understanding the function of the CYP450 gene family.

## Materials and methods

### Plant materials

T2T genome sequence of wild *P. grandiflorus* was collected from Tongcheng City, Anhui Province, China (30.983863°N, 116.742667°E). Fresh tissues were separated and immediately frozen in liquid nitrogen. Samples from different growth stages (May, August, and October) of *P. grandiflorus* were collected, and fresh roots were used for metabolite detection and transcriptome sequencing (three biological duplicates per month).

### Genome survey

The genome size of *P. grandiflorus* was estimated using k-mer analysis. Approximately 34 889 Mb of filtered clean reads was used to calculate the 19-mer frequency distribution using Jellyfish v2.2.10 [49]. Genome size and heterozygosity were computed using the GenomeScope v2.0 [50].

### DNA extraction

High-quality genomic DNA was extracted using a TIANGEN Kit (TIANGEN BIOTECH [BEIJING] Co., Ltd.). DNA quality and concentration were evaluated using 0.75% agarose gel electrophoresis, and its purity was assayed using a NanoDrop One spectrophotometer (Thermo Fisher Scientific) and Qubit 3.0 Fluorometer (Life Technologies, Carlsbad, CA, USA).

### Genome sequencing

Genome sequencing was performed using Nanopore Ultra-long, PacBio HiFi, Hi-C, and next-generation sequencing. Sequencing was performed by Wuhan Benagen Technology Co. Ltd. (China). Ultra-long sequencing was performed using the Nanopore PromethION platform, and the raw data were filtered to remove failed reads with an average quality value of <7. Fragments shorter than 10 kb were trimmed using Filtlong software (v0.2.4, https://github.com/rrwick/Filtlong), and adaptor sequences were clipped using Porechop v0.2.4 (https://github.com/rrwick/Porechop) [51]. The obtained reads were subjected to final filtration (length < 30 kb, mean read quality score ≤ 90%) and used for genome assembly. The HiFi raw data sequenced via the Pacbio Revio platform were filtered by CCS v6.0.0 (https://github.com/PacificBiosciences/ccs) with the following parameters: min-passes 3, min-snr 2.5, and top-passes 60). The obtained CCS (circular consensus sequencing) reads obtained were prepared for subsequent analyses [52]. Hi-C and next-generation sequencing were used for auxiliary assembly and annotation. The Hi-C raw data were trimmed using fastp v0.21.0, and then aligned to the reference genome using HICUP v0.8.0 (http://www.bioinformatics.babraham.ac.uk/projects/hicup). Raw reads obtained from next-generation sequencing were filtered using fastp v0.21.0, to remove reads of low quality, short length, high N-base content, and adaptor contamination. Repeat sequences generated from PCR amplification were also eliminated.

### T2T genome assembling and telomere repair

For ONT ultra-long read assembly, NextDenovo v2.5.0 (https://github.com/Nextomics/NextDenovo) was first used for preliminary data processing with the parameters read_cutoff = 1 k, blocksize = 1 g, and nextgraph_options = −a1. HiFi reads were assembled into contigs using Hifiasm v0.18.2 [53]. Minimap2 v2.17-r941 was used to identify bacterial contamination and contigs using low-read mapping [54]. Based on the Hi-C interaction relationship, contigs were clustered and oriented using ALLHIC v0.9.8. The correct contigs were then converted to input files using 3D-DNA v180419 and manually ordered using Juicebox v1.11.08 [55–57]. After removing the heterozygous sequences, gaps were filled with 100 N to generate the final chromosome-scale genome sequence. The reliability of gap-filling was validated using different versions of assembled reads and ONT or HiFi read mapping to alternative regions. Contigs were anchored to chromosomes, and the interaction intensity was plotted using HiCExplorer v3.6 [58].

Telomeres are complex nucleoprotein structures consisting of repetitive DNA sequences at the ends of chromosomes [59]. Based on the telomere database (http://telomerase.asu.edu/sequences_telomere.html), telomeric repeat units, 5′-CCCTAAA and 3′-TTTAGGG, were detected per read and reassembled to generate consensus sequences. The consensus sequence was mapped to each chromosome and the terminal telomere region was replaced by mapping identity ≥80 using nucmer v3.1 [60].

### Genome quality assessment

The continuity of the genome assembly was evaluated according to the position and number of gaps. Genomic consistency was estimated by mapping secondary, tertiary, and HiFi sequencing data to the genome and calculating the mapping and coverage rates. Its integrity and exactitude were assessed using BUSCO v5.3.0 (−evalue 1e-05) and k-mer statistics.

### Repetitive element identification

Repeat sequences were predicted using RepeatMasker v4.1.5 (parameters: -noLowSimple -p-value 0.0001) based on two libraries: RepBase (version 20 181 026) and de novo repeat libraries. A de novo repeat library was constructed using RepeatModeler v2.0.4, to predict homologous sequences, and LTR_FINDER (official release of LTR_FINDER_parallel) and LTRharvest v4.62, to predict LTR [61,62]. The LTR_retriever v2.1.3 was used to remove redundant LTR sequences. TE proteins were identified using RepeatProteinMask v4.1.5. All the above predictions were combined, and redundancy was removed to obtain the final repetitive elements.

### Gene prediction and functional annotation

Genes were predicted using the following three approaches: homology search, de novo prediction, and transcriptome sequencing. Homologous proteins from *Arabidopsis thaliana*, *Codonopsis lanceolata*, *Helianthus annuus*, and *Panax ginseng* were aligned to the genome to generate candidate regions. The software Exonerate v2.4.0, was applied to forecast protein-coding genes [63]. For de novo prediction, Augustus v3.5.0 was used to predict ab initio genome models [64]. Based on RNA-sequencing prediction, transcriptome data were mapped to the genome using Hisat2 v2.2.1, and Minimap2 v2.26-r1175, and transcripts were reassembled using Stringtie v2.2.1 [65,66]. All the predicted gene sets were integrated and verified to obtain the final dataset. For functional annotation, all protein-coding genes were aligned to UniProt (20220309), NR (20220309), GO (20220309), Pfam (20230311), Interpro (5.55–88.0), and KEGG (20230711). In addition, tRNA and rRNA were separately identified using tRNAscan-SE v2.0.12 and RNAmmer v1.2 [67,68]. snRNAs and miRNAs were predicted by searching Rfam (14.9) using INFERNAL v1.1.4 [69].

### Collinearity analysis of different genomes

T2T *P. grandiflorus* genome was aligned to the previous version of genome assembly, which was obtained from National Genome Data Center (https://ngdc.cncb.ac.cn/search/?dbId=gwh&q=GWHARYT00000000.1) using Mummer v4.0.0. Syntenic regions were identified using Syri v1.6, and constructive rearrangements were compared between the two genomes [70].

### Identification of centromeres

Centromeres are primarily composed of centromeric tandem duplications, retrotransposons, and low-copy sequences. Candidate centromeric tandem repeats were identified using Tandem Repeats Finder and were continuously clustered in each chromosome using cd-hit [71,72]. Tandem repeat monomers with similarities >90% were assigned to one cluster, and the top nine abundant tandem repeat clusters (TRCs) were plotted. Using RepeatMasker, we set the longest tandem repeat cluster as a query and searched for it on each chromosome to reduce the overlap among TRCs. The locations of the centromeric regions were estimated by merging all TRCs regions, and rectified by targeting next-generation sequencing data using the k-mer anchoring method.

### Gene family identification and phylogenetic analysis

Twelve species—*A. thaliana*, *A. lappa*, *C. sinensis*, *C. sativa*, *C. lanceolata*, *D. carota*, *H. annuus*, *L. sativa*, *O. sati*va, *P. ginseng*, *S. lycopersicum*, and *V. vinifera*—were included in the genome evolution analysis. Orthofinder v2.3.12 was used for gene family clustering [73]. The identified single-copy genes were used to construct the phylogenetic tree. Common and specific gene families were functionally annotated by searching the KEGG and GO databases [74,75]. Multiple sequence alignments of single-copy gene families were performed using the Muscle v3.8.31 [76]. The alignment was trimmed using Trimal v1.2rev59 with the parameter -gt 0.2, and then merged and input into the RAxML v8.2.10 software for ML tree construction [77]. The gene family and contraction expansion in each evolutionary branch were assessed using CAFE v3.1. Significant expansion and contraction of gene families were defined as *p* ≤ 0.05 [78].

### Comparative genomics analysis

To estimate the divergence time of each phylogenetic tree node, the Mcmctree program of PAML v4.9 was applied based on fossil records (http://www.timetree.org) pursuant to the following parameters: nsample = 3 000 000, burnin = 8 000 000, seqtype = 0, model = 4 [79]. Collinearity analysis among the species was performed using JCVI v0.9.13 [80,81]. Ks values of homologous gene pairs are commonly used to predict WGD events. Syntenic blocks were identified using MCScanX, and Ks_Ka values were calculated using the yn00 module of PAML v4.9 [82]. The Ks distribution was plotted using ggplot2 v2.2.1. Furthermore, the protein sequences of single-copy gene families were aligned and back-translated into codons using PAL2NAL [83]. The CodeML module of PAML was used for positive selection analysis based on the branch site model. Based on the two models (model A and null model), the chi2 program was implemented for likelihood ratio tests to obtain significantly positively selected genes (*p* < 0.5).

### Genome mining for genes involved in triterpenoid saponins biosynthesis

Two methods were combined to identify genes related to triterpenoid biosynthesis: KEGG database annotation and a homologous protein BLAST search. The Kofam domain database, which belongs to the KEGG database, was used for the functional annotation of genes according to motif similarity [84]. KEGG annotation yielded KEGG pathway maps and genes that hit specific pathways. We focused on two relevant pathways: map00900 (terpenoid backbone biosynthesis) and map00909 (sesquiterpenoid and triterpenoid biosynthesis). Furthermore, we downloaded the protein sequences involved in triterpenoid biosynthesis from other species, and used these proteins as queries for a homologous BLAST search against the genes of the T2T genome (cutoff of 1e-50). All results were merged into the initial gene set. It then underwent redundancy removal, open reading frame verification, and domain analysis using the conserved domain database (CDD) of the NCBI to generate final full-length coding genes [85].

### Genome-wide identification of the cytochrome P450 superfamily

An HMM of P450.hmm (PF00067) was downloaded from the Pfam database (version 36.0) and was used as the query to execute the search against the *P. grandiflorus* T2T genome using HMMER v3.3.2 (cutoff of 1e-5). According to gene annotations from the Pfam and KEGG databases, genes encoding CYP450 were extracted as candidates. In parallel, CYPs from *A. thaliana*, *Polygala tenuifolia*, and *Medicago truncatula* were retrieved from NCBI, and their amino acid sequences were used as queries to perform a BLASTP search in the T2T protein database (cutoff of 1e-50). All yielded sequences with protein lengths <300 aa were eliminated and subjected to NCBI CDD (https://www.ncbi.nlm.nih.gov/Structure/cdd/wrpsb.cgi) and ORF Finder (https://www.ncbi.nlm.nih.gov/orffinder/) tools for further validation. The final non-redundant CYPs were identified as the CYP gene families. MEGA v11 was used to perform protein alignment using the ClustalW algorithm and to construct an NJ tree with 1000 bootstrap replicates [86].

### Transcriptome analysis of *P. grandiflorus* roots at different growing stages

Transcriptome sequencing was performed using the DNBSEQ platform. Raw RNA reads were filtered and trimmed to yield clean reads, which were then mapped onto the T2T reference genome. The fragments per kilobase of exon per million fragments (FPKM) of the genes were calculated according to the sequencing depth and gene length. The expression levels of these genes were analyzed and visualized using TBtools based on the FPKM values [87].

### Determination of metabolites in *P. grandiflorus* roots under different growing stages

The root tissues were freeze-dried, and each individual was precisely weighed to 50 mg and subsequently extracted with 1200 μL of a precooled methanol:H_2_O mixture (7:3) by vortexing six times at 30-minute intervals. The extracts were centrifuged (12 000 rpm, 3 minutes), and the supernatants were assayed using QTRAP™ 4500 LC–MS/MS. The assay was performed using an ultra-performance liquid chromatography (UPLC) ExionLC™ AD (SCIEX, Framingham, MA, USA) coupled with tandem mass spectrometry (MS/MS) (SCIEX, Framingham, MA, USA). Analytes were separated with an Agilent SB-C18 column (1.8 μm, 2.1 mm × 100 mm), using 0.1% formic acid-water (A) and 0.1% formic acid-acetonitrile (B) as the mobile phase at a flow rate of 0.35 mL/min. The gradient elution program was as follows: 0–5 minutes, 5–95% B; 9–10 minutes, 95% B; 10–11 minutes, 95–5% B; 11–14 minutes, 95% B. The column temperature was maintained at 40°C, and the injection volume was 4 μL. The mass spectrometer was operated in positive or negative ion mode using multiple reaction monitoring. Metabolomic analyses were performed using Metware Biotechnology, Inc. Considering that metabolomics has limitations in detecting high-molecular-weight components, we used UPLC-Orbitrap-MS/MS to determine the content of platycodins.

## Supplementary Material

Web_Material_uhaf030

## Data Availability

The T2T genome assembly and RNA-seq data of P. grandiflorus were deposited in the National Genome Data Center, under the accession numbers PRJCA031148 and PRJCA026736. The genome data referred in this article: *Arabidopsis thaliana* (NCBI: GCF_000001735.4), *Arctium lappa* (NCBI: GCA_023525745.1), *Camellia sinensis* (NGDC: GWHASIV00000000), *Daucus carota* (NGDC: GCA_030127425.1), *Cannabis sativ*a (NGDC: GWHABGK00000000), *Helianthus annuus* (NCBI: GCA_026651805.1), *Lactuca sativa* (NGDC: GCF_002870075.4), *Oryza sativa* (NCBI: GCF_001433935.1), *Panax ginseng* (NGDC: GWHBEIL00000000.1), *Solanum lycopersicum* (NCBI: GCA_915070445.1), *Vitis vinifera* (NCBI: GCF_030704535.1), Codonopsis lanceolata (https://figshare.com/articles/dataset/First_Report_of_Chromosome-Level_Genome_Assembly_for_Lance_Asiabell_Codonopsis_lanceolata_A_Medicinal_and_Vegetable_Plant_in_the_Campanulaceae_Family/21507774?file=38116599).
