# Supplementary material for: Telomere-to-telomere gap-free genome assembly provides genetic insight into the triterpenoid saponins biosynthesis in Platycodon grandiflorus
Source: Hortic Res. 2025 Feb 1;12(5):uhaf030. doi: 10.1093/hr/uhaf030 (PMC11992332; doi:10.1093/hr/uhaf030)
Supplement: Web_Material_uhaf030 [file web_material_uhaf030.zip › Supplementary figure1-24.pdf]

Supplementary Figures

A telomere-to-telomere gap-free genome assembly provides genetic insight into the triterpenoid saponins biosynthesis in *Platycodon grandiflorus*

Hanwen Yu[1], Haixia Wang[1], Xiao Liang[1], Juan Liu[2], Chao Jiang[2], Xiulian Chi[2], Nannan Zhi[1], Ping Su[2,*], Liangping Zha[1,3,4,5,*], Shuangying Gui[1,4,6,7,*]

[1] College of Pharmacy, Anhui University of Chinese Medicine, Hefei, 230012, China
[2] State Key Laboratory for Quality Ensurance and Sustainable Use of Dao-di Herbs, National Resource Center for Chinese Materia Medica, China Academy of ChineseMedical Sciences, Beijing, China
[3] Institute of Conservation and Development of Traditional Chinese Medicine Resources, Anhui Academy of Chinese Medicine, Hefei 230012, China
[4] MOE-Anhui Joint Collaborative Innovation Center for Quality Improvement of Anhui Genuine Chinese Medicinal Materials, Hefei 230012, China
[5] Center for Xin'an Medicine and Modernization of Traditional Chinese Medicine of IHM, Anhui University of Chinese Medicine, Hefei 230012, China
[6] Institute of Pharmaceutics, Anhui Academy of Chinese Medicine, Hefei, China
[7] Anhui Province Key Laboratory of Pharmaceutical Preparation Technology and Application, Hefei, China
* Corresponding authors: Ping Su, suping120@163.com; Liangping Zha, zlp_ahtcm@126.com; Shuangying Gui, guishy0520@126.com

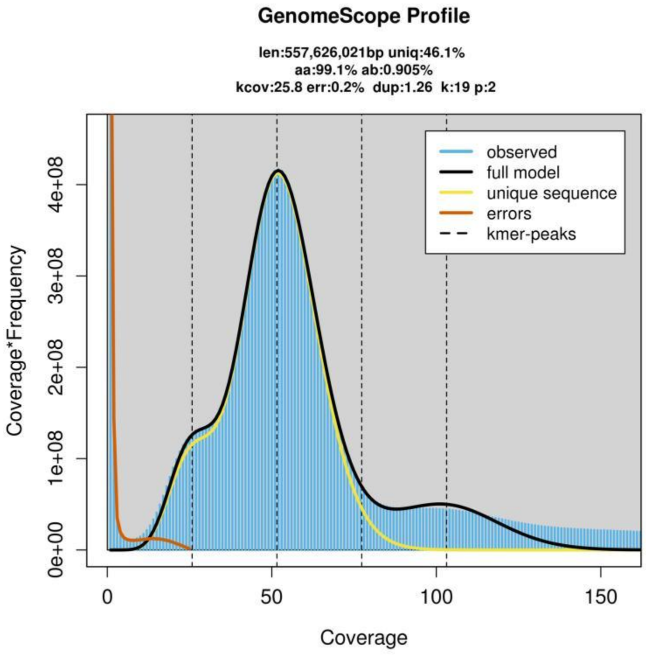

Figure S1. GenomeScope profile of the *P. grandiflorus* genome inferred using a k-mer size of 19.

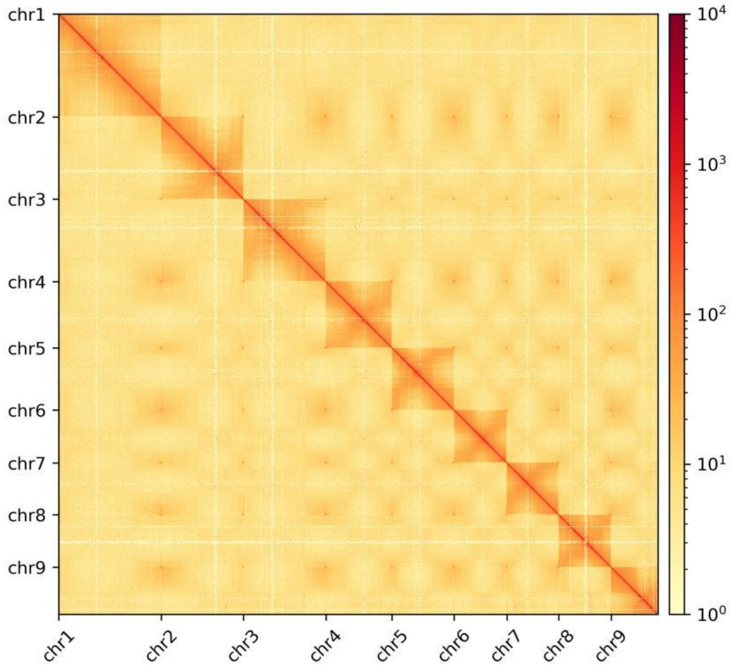

Figure S2. Hi-C heatmap of chromosome interactions.

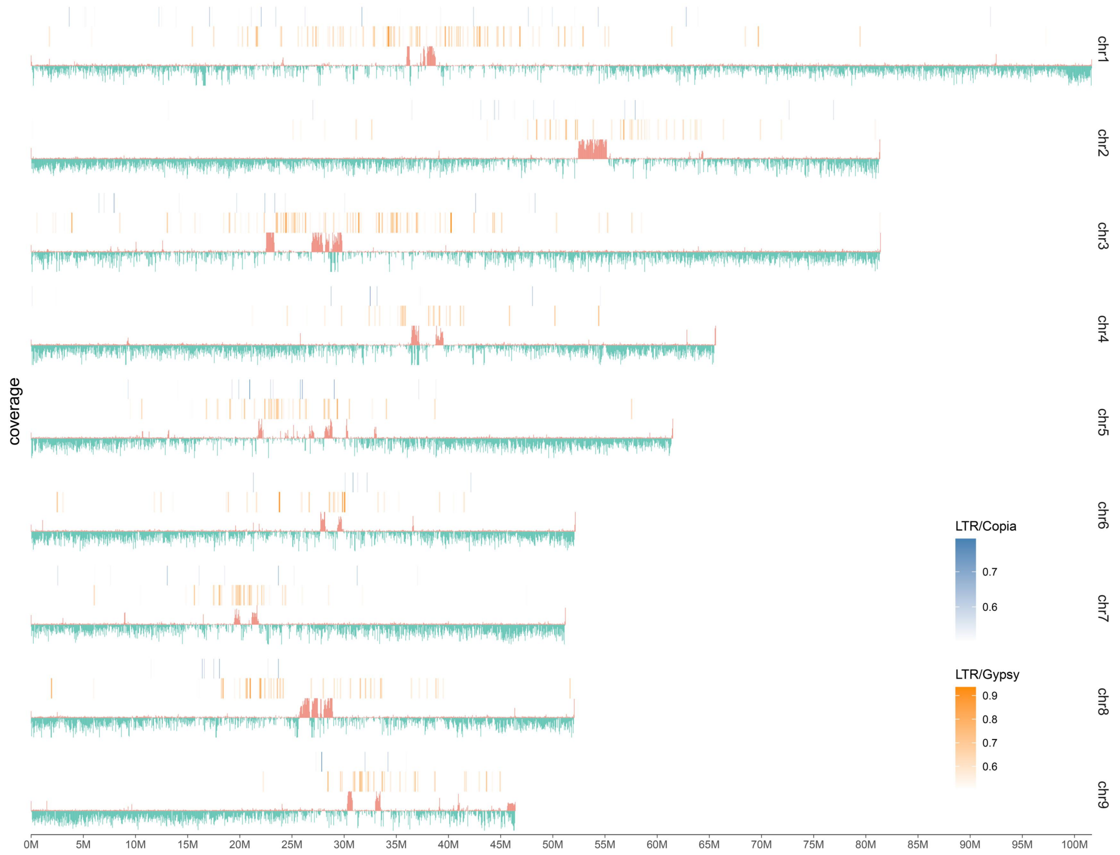

Figure S3. The plot of LTR, tandem repeat and gene densities. Blue and yellow strips represent *Copia* and *Gyspy*. Brick-red strip represent tandem repeat density, and green strip represent gene density. The plot is drawn by bedtools v2.30.3, and the coverage of tandem repeat and gene is calculated in 50 kb windows.

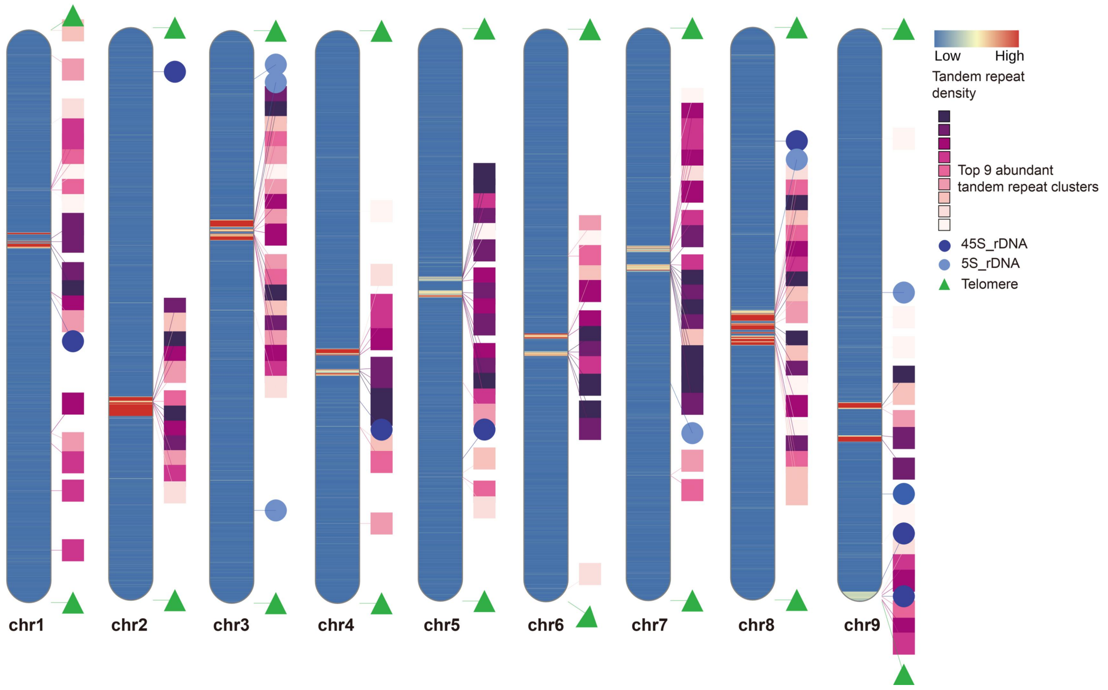

Figure S4. The location of top 9 abundant tandem repeat clusters in each chromosome. The abundance of 9 clusters is shown by color depth.

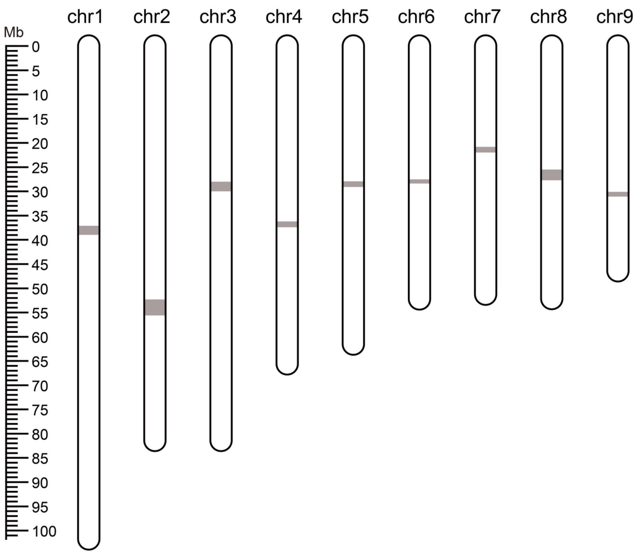

Figure S5. 9 centromeric regions in *P. grandiflorus* T2T genome.

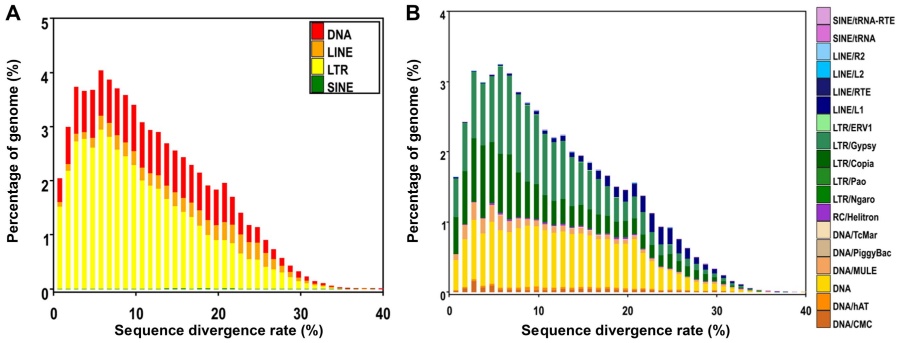

Figure S6. Transposons identified in *P. grandiflorus* genome. (A) Plot of transposons divergence. (B) Plot of detailed transposons subtypes divergence.

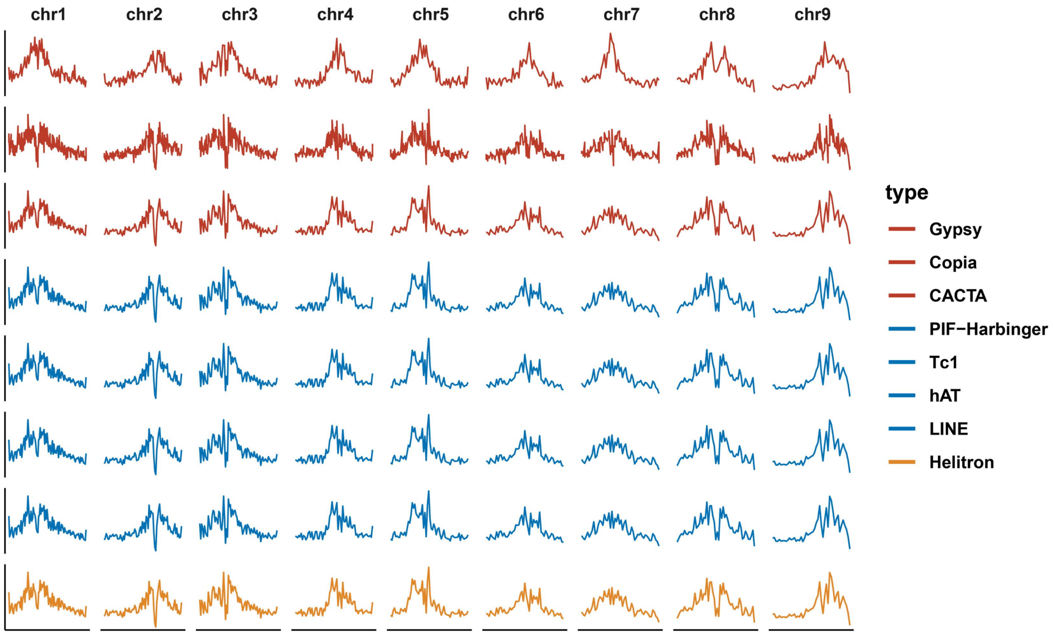

Figure S7. Transposons distribution on the 9 chromosomes.

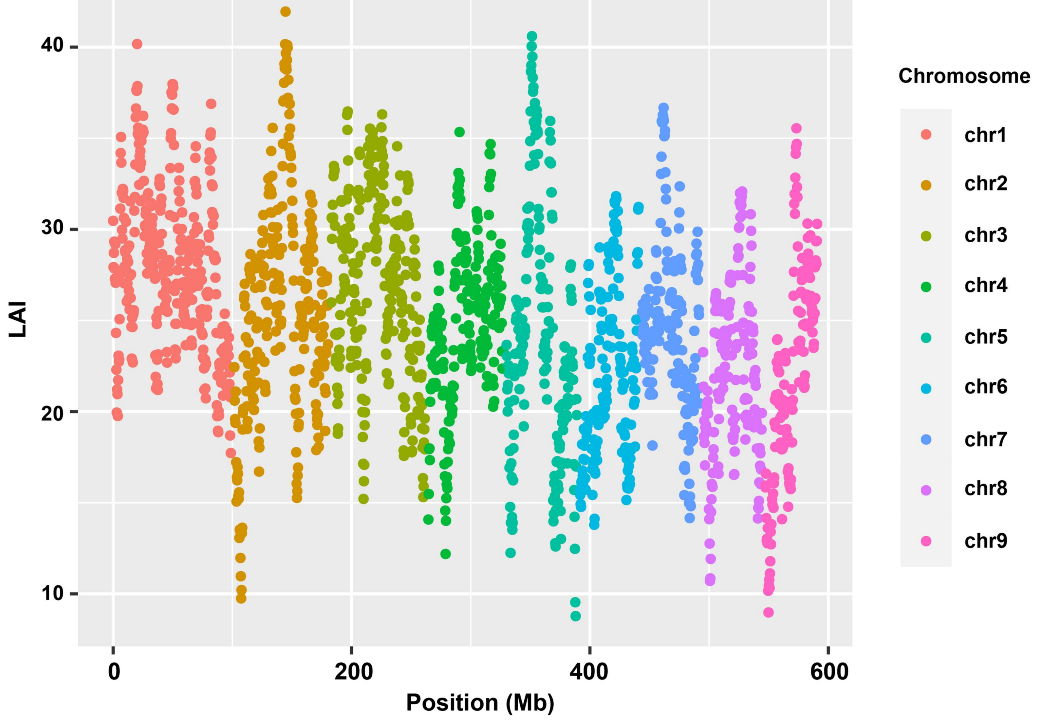

Figure S8. LAI assessment for each chromosome of *P. grandiflorus* T2T genome. The average LAI is 25.83.

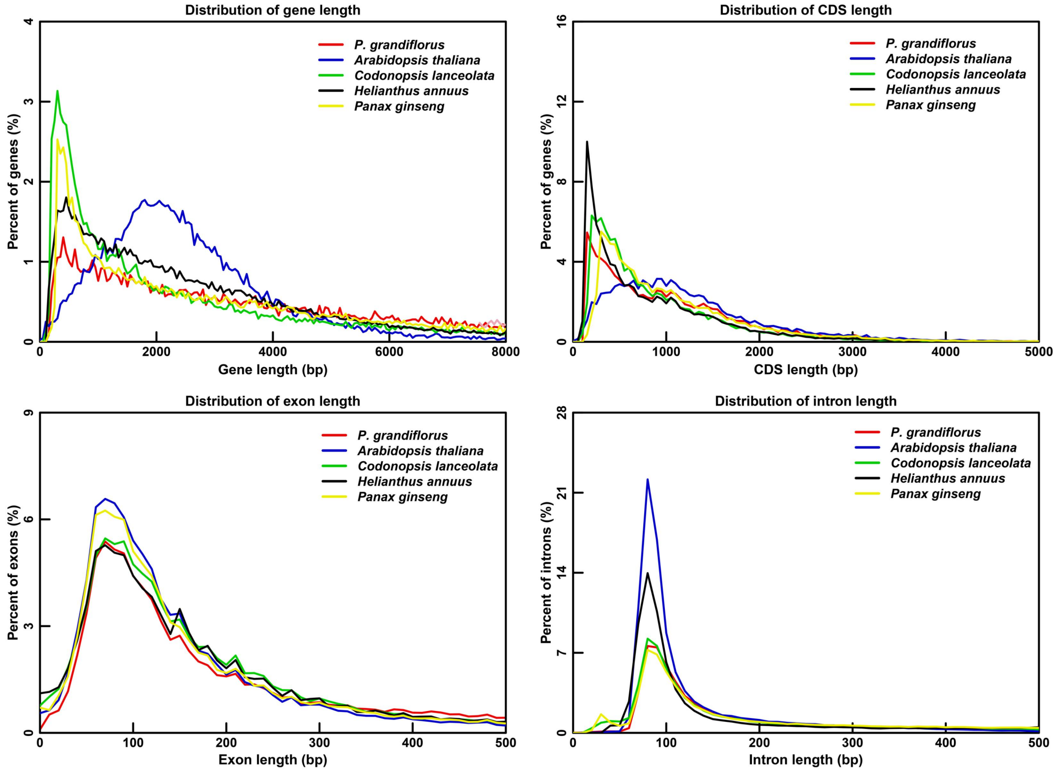

Figure S9. Gene prediction of *P. grandiflors* genome and other species.

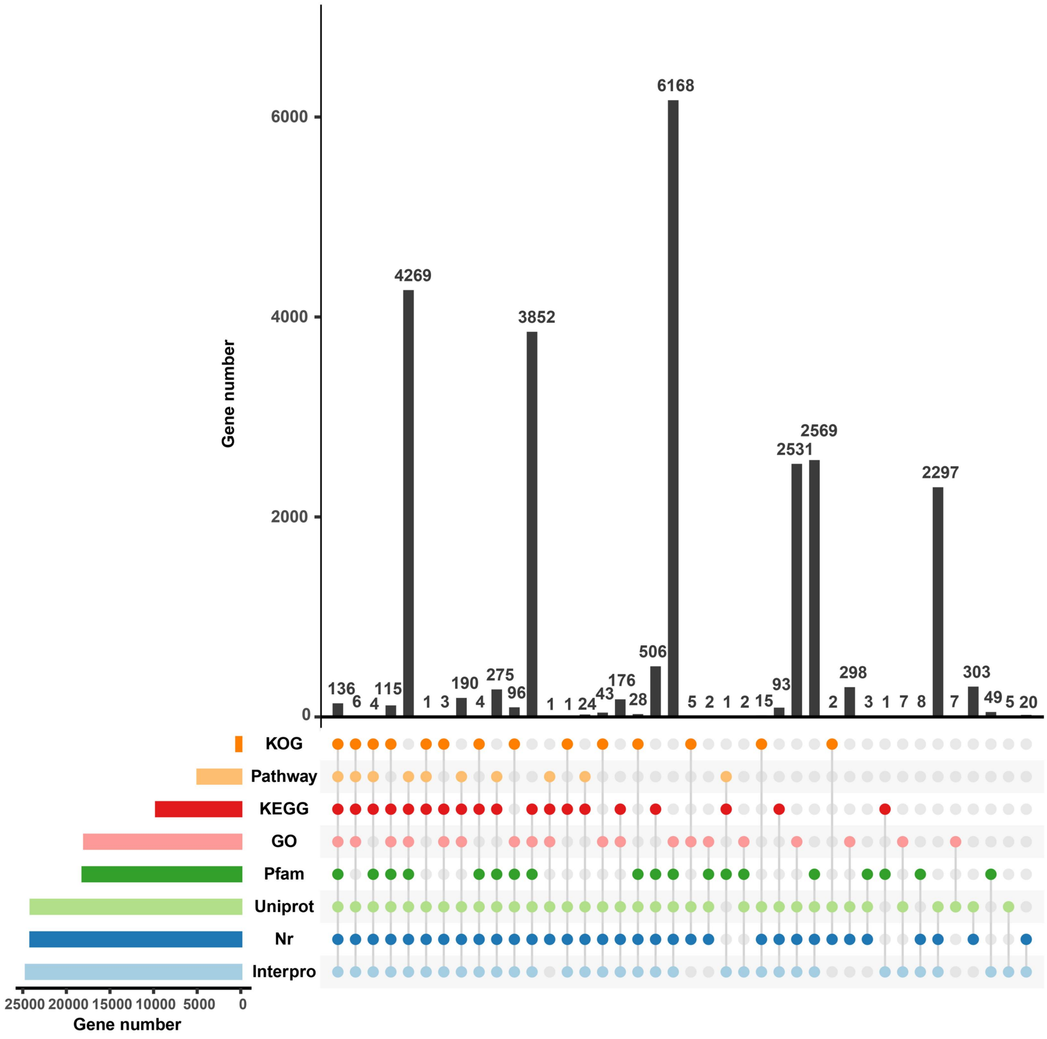

Figure S10. Annotation of predicted genes in different databases.

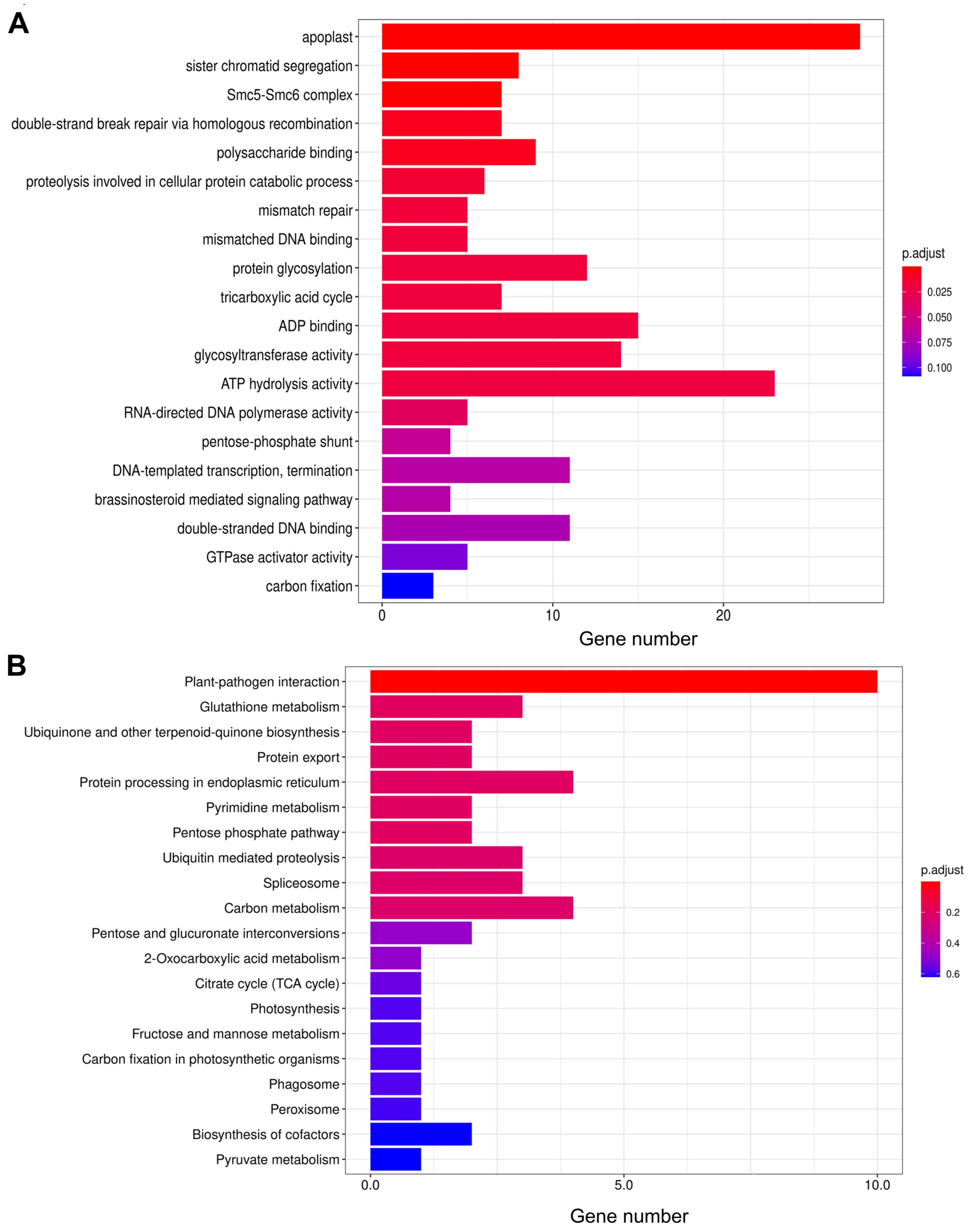

Figure S11. GO and KEGG enrichment of uniq gene families in *P. grandiflorus*. (A) GO annotation. (B) KEGG annotation. Adjusted *p* value was displayed via color.

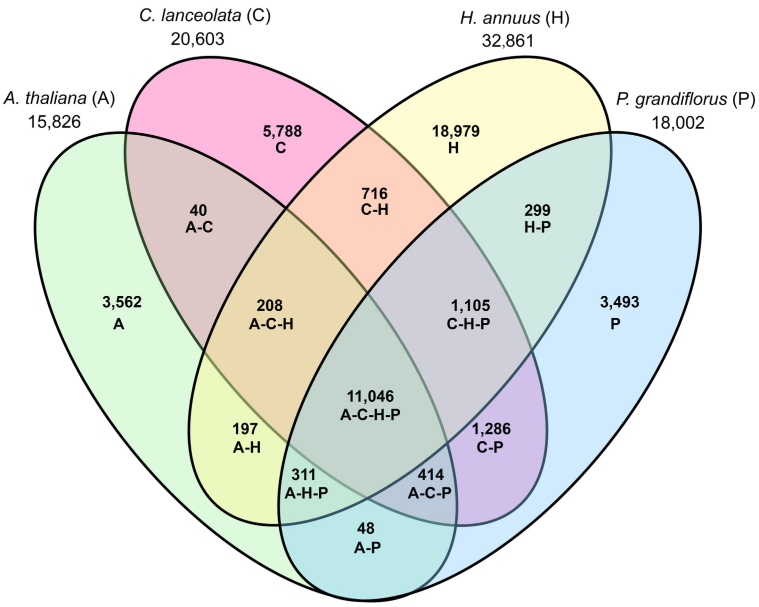

Figure S12. Venn diagram of gene families in *P. grandiflorus*, *C. lanceolata*, *H. annuus*, and *A. thaliana*.

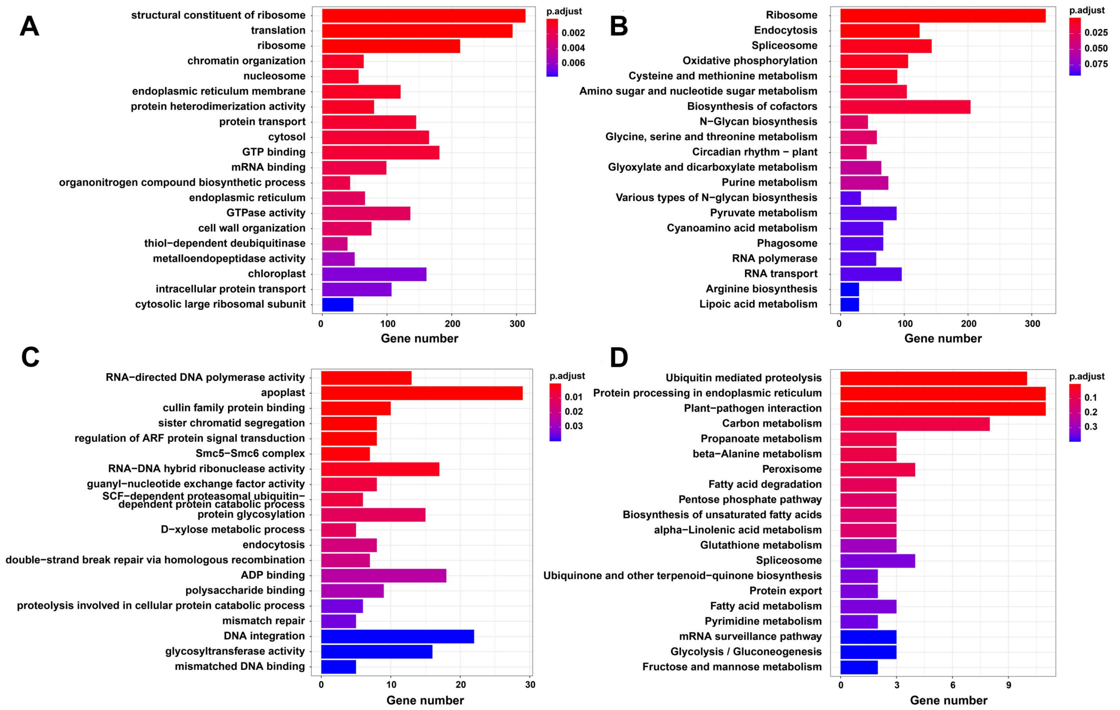

Figure S13. GO and KEGG annotation of shared and unique gene families in *P. grandiflorus*, *C. lanceolata*, *H. annuus*, and *A. thaliana*. (A) GO enrichment of shared gene families in four species. (B) KEGG enrichment of shared gene families in four species. (C) GO enrichment of unique gene families in *P. grandiflorus*. (D) KEGG enrichment of unique gene families in *P. grandiflorus*. Adjusted *p* value was displayed via color.

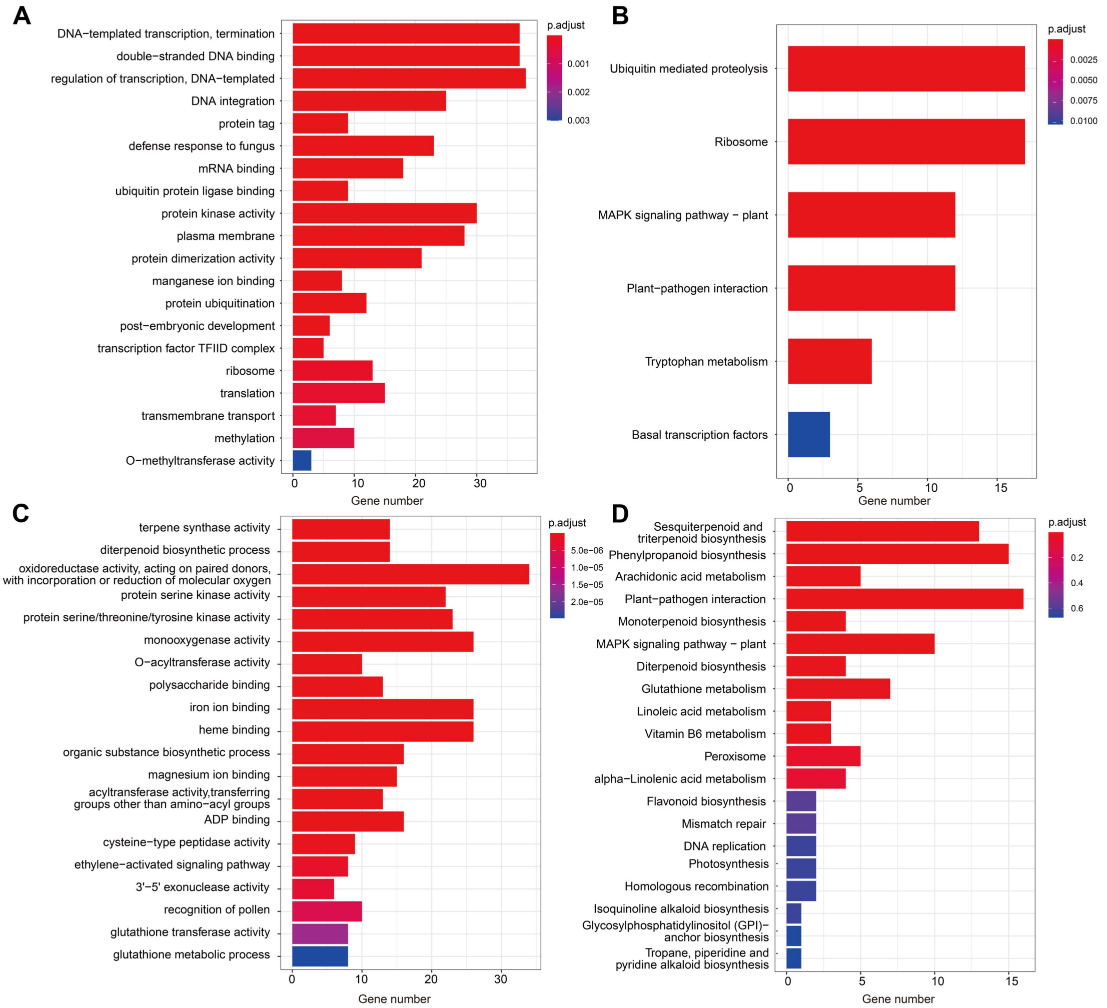

Figure S14. GO and KEGG annotation of expanded and contracted gene families in *P. grandiflorus*. (A) GO enrichment of expanded genes. (B) KEGG enrichment of f expanded genes. (C) GO enrichment of f contracted genes. (D) KEGG enrichment of contracted genes. Adjusted *p* value was displayed via color.

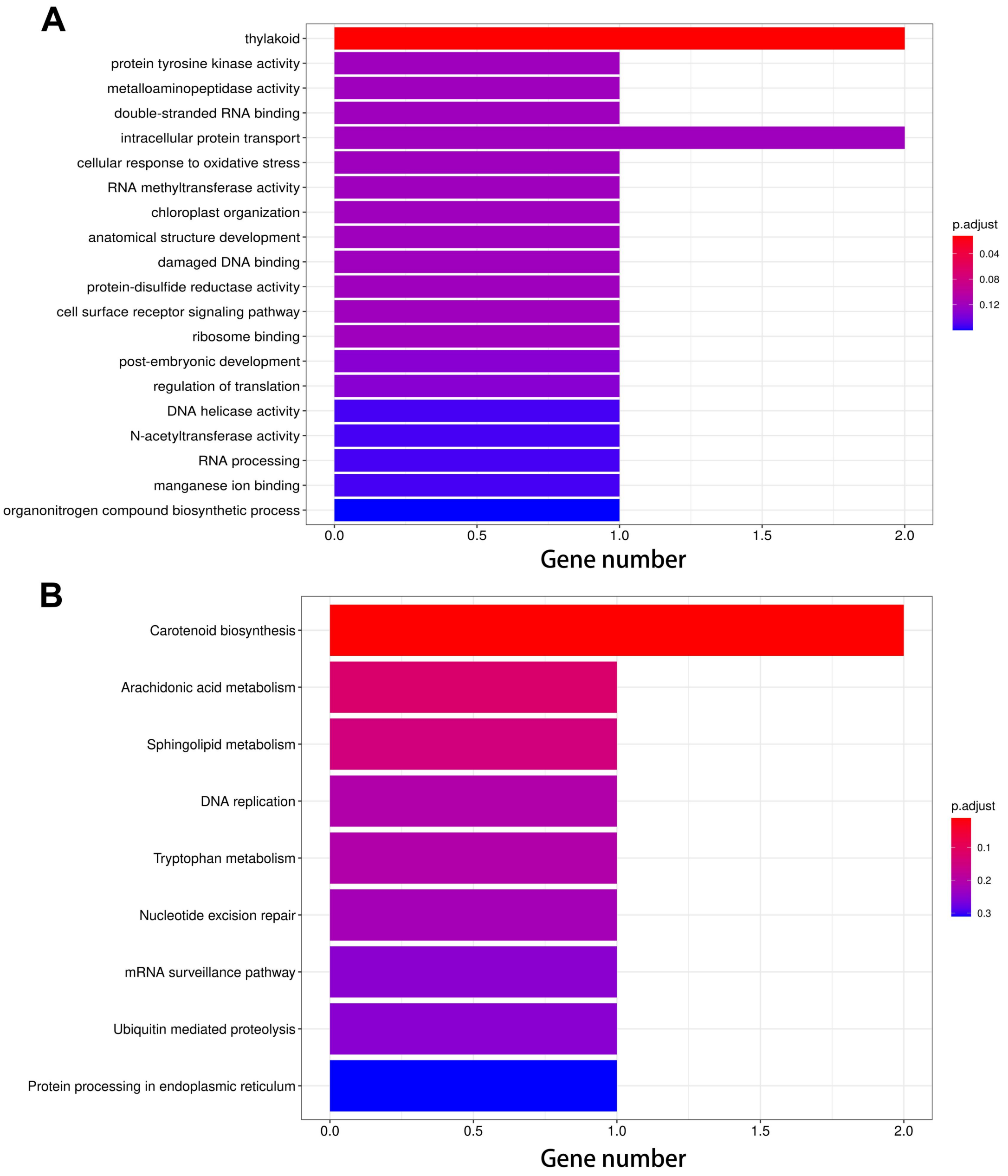

Figure S15. GO (A) and KEGG annotation (B) of significant positive selected genes. Adjusted *p* value was displayed via color.

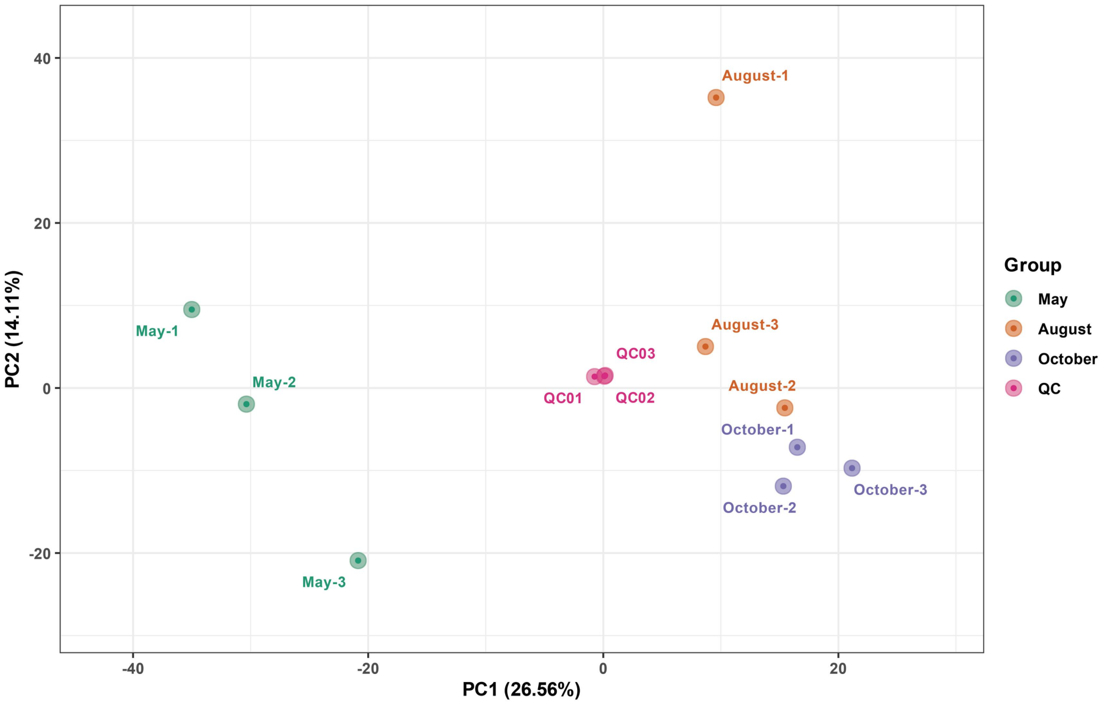

Figure S16. PCA of all metabolites in each sample.

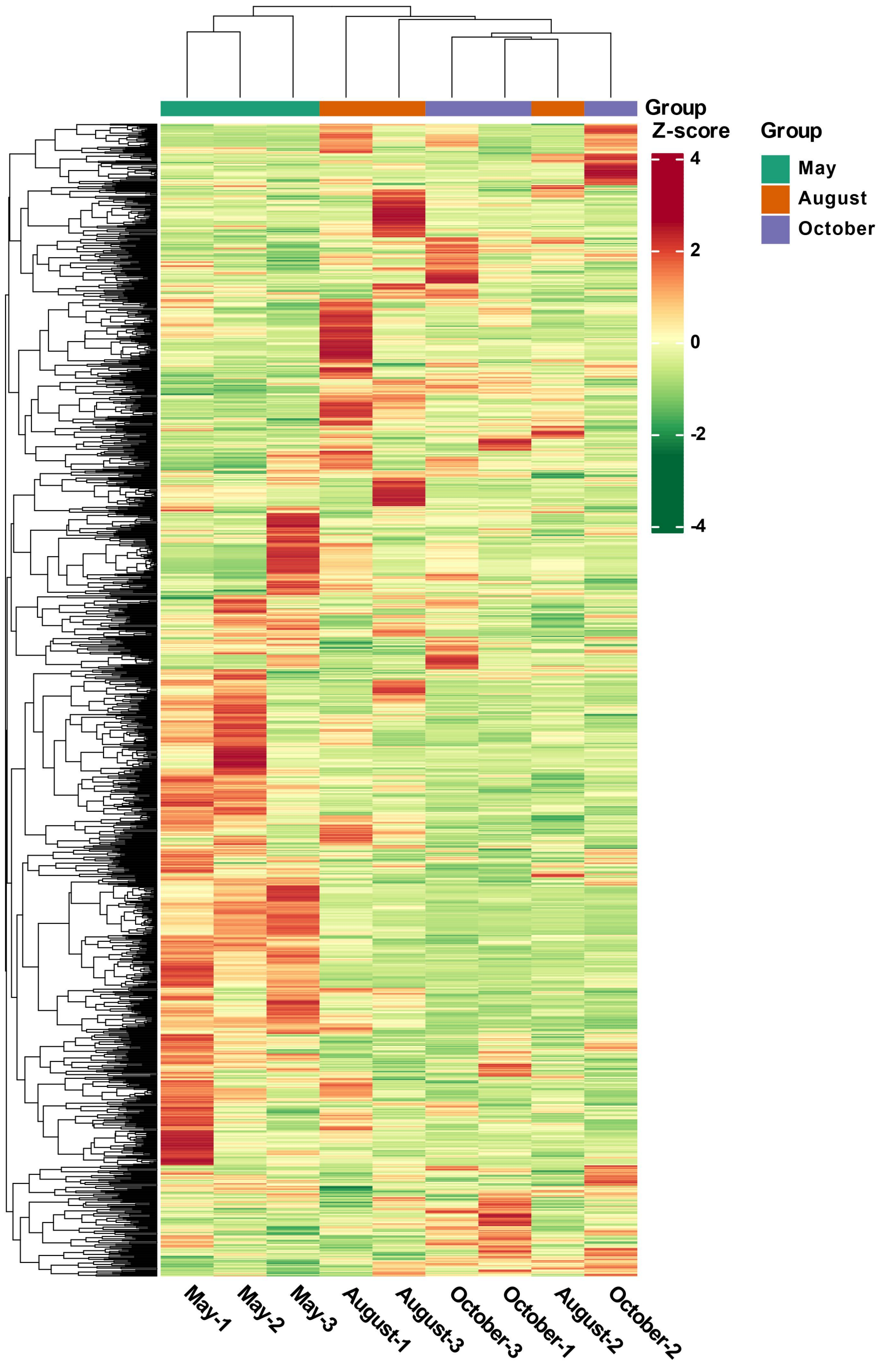

Figure S17. Heatmap clusters of all types of metabolites in each sample. The values were normalized and each metabolite is displayed in a row.

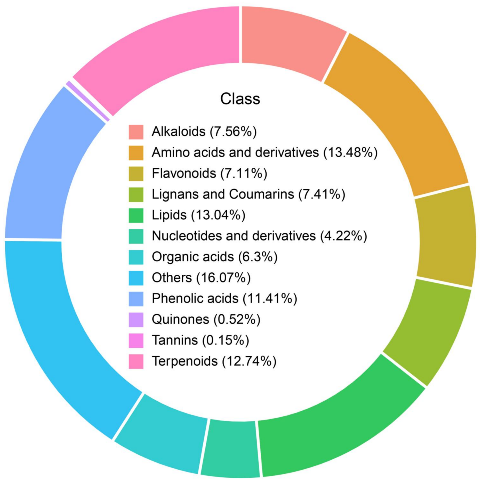

Figure S18. Classes of identified 1,350 metabolites of *P. grandiflorus* roots.

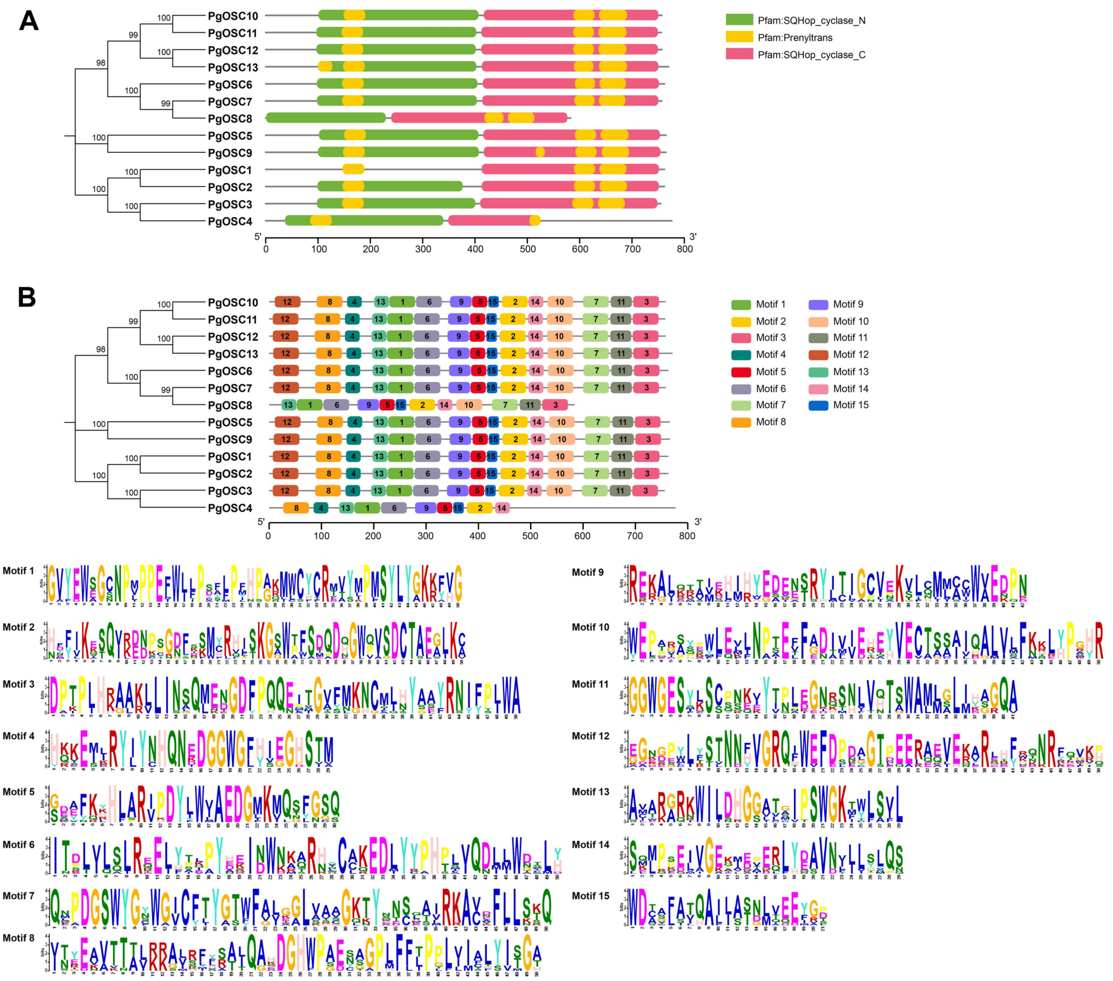

Figure S19. Domain structure (A) and motif compositions (B) of PgOSC proteins.

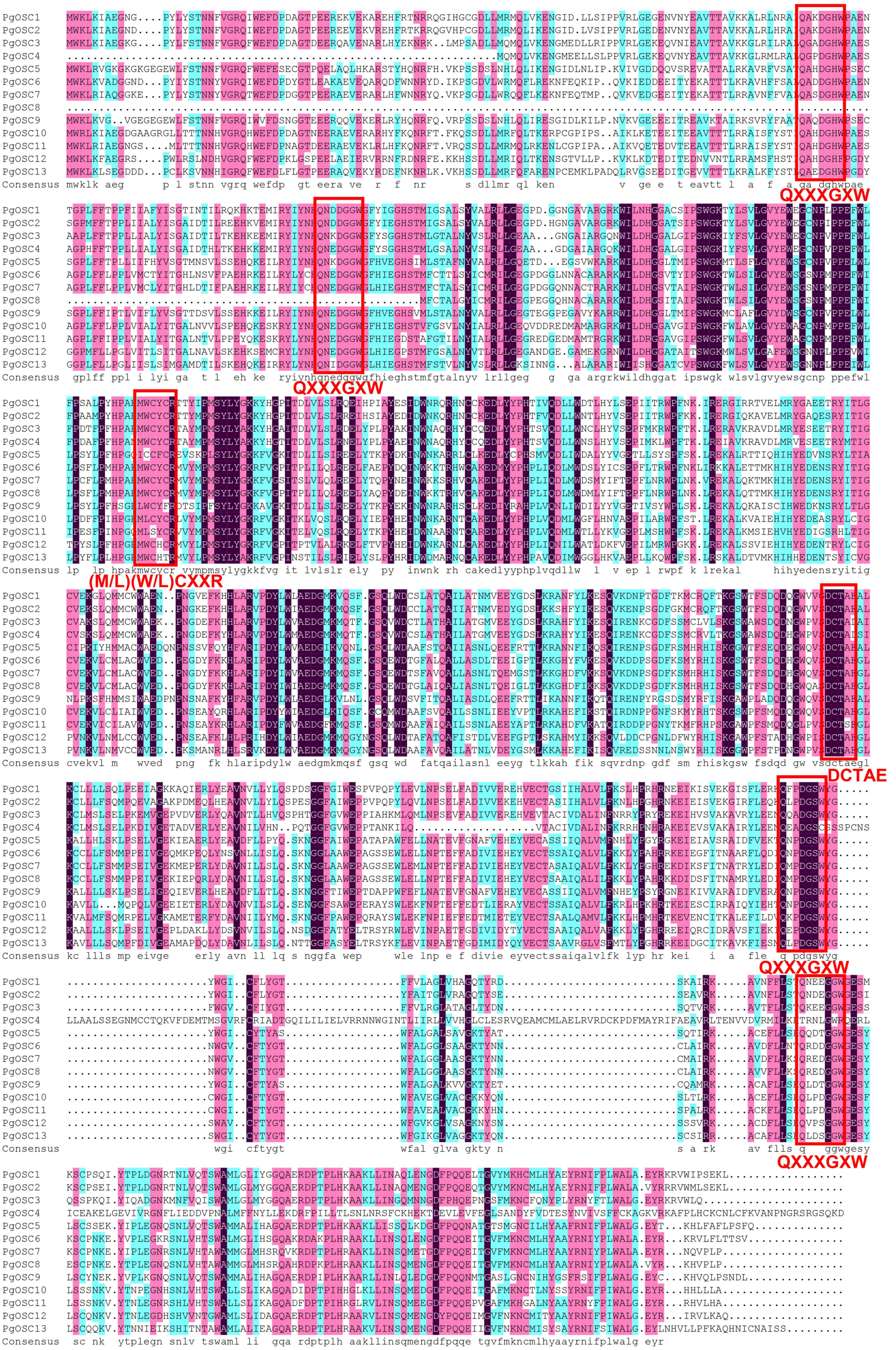

Figure S20. Multiple sequence alignment of candidate PgOSCs. The alignment was generated with DNAMAN. Conserved motifs are denoted by boxes.

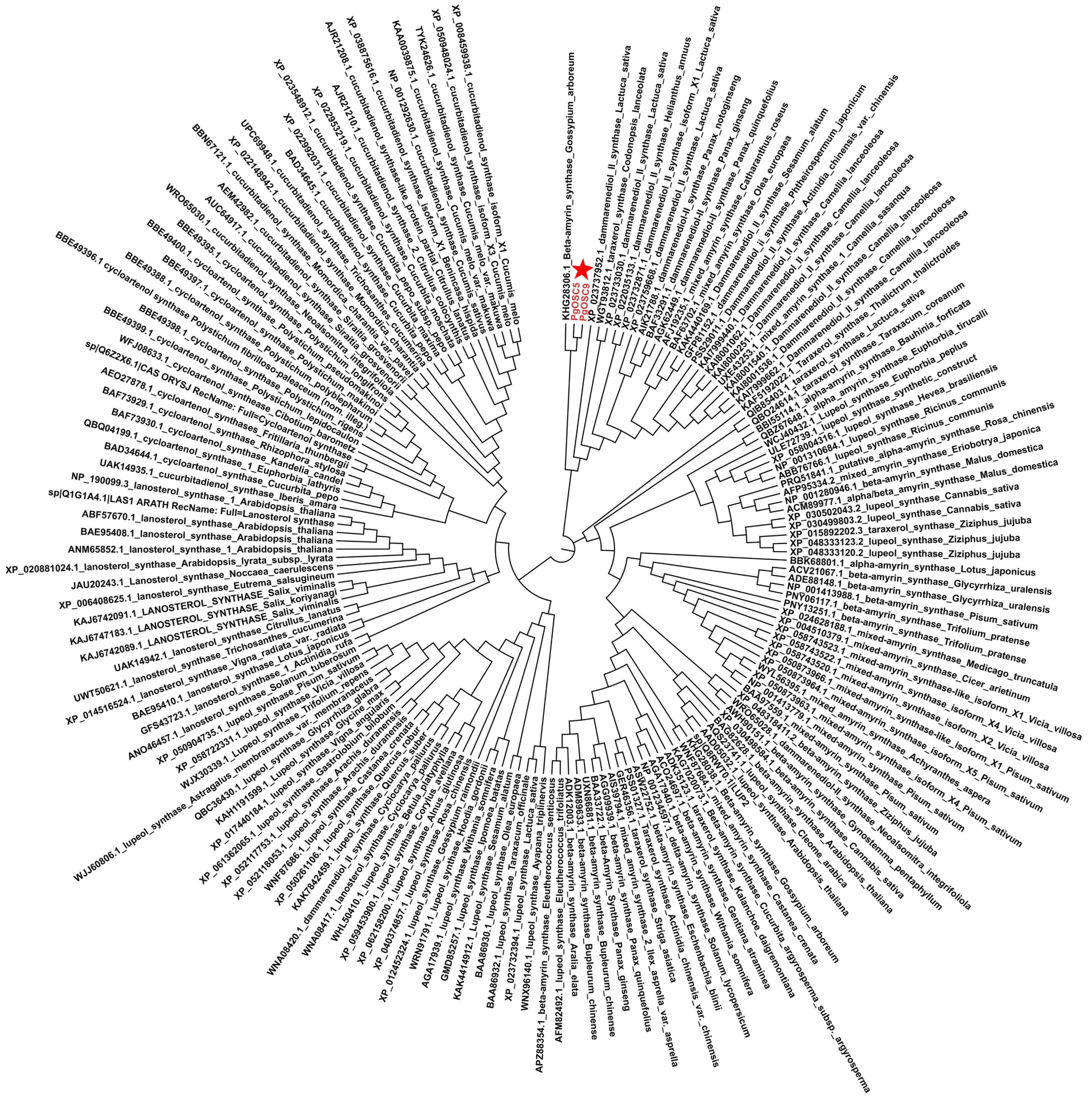

Figure S21. A maximum-likelihood tree construction of PgOSC5, PgOSC9 and other plant OSC proteins.

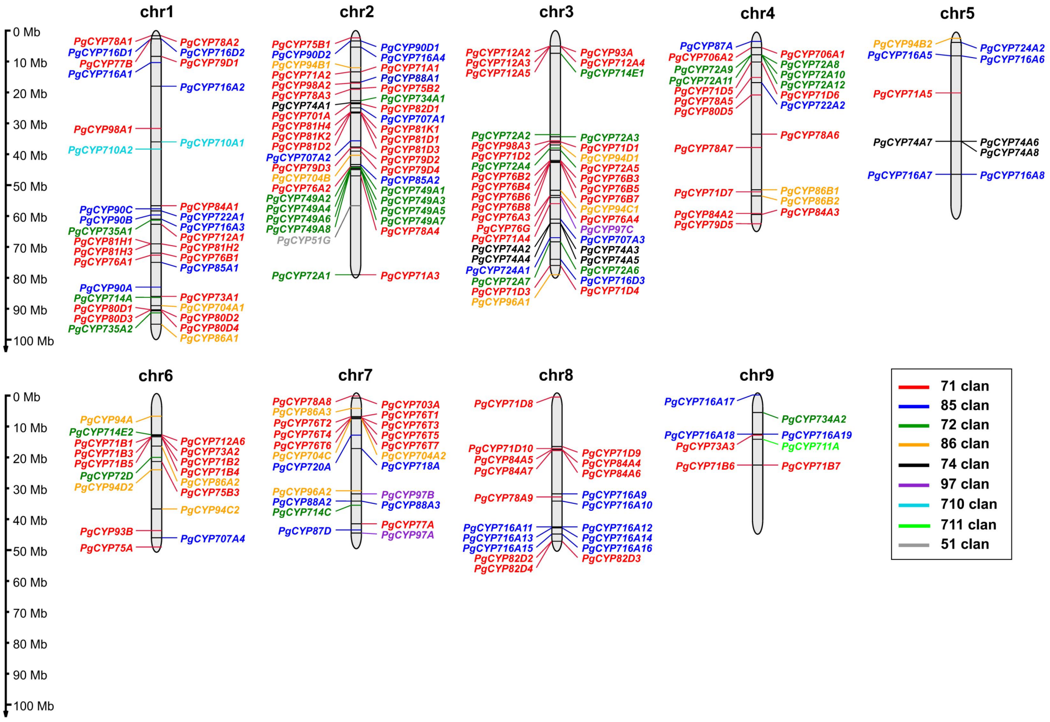

Figure S22. Chromosomal mapping of 211 *PgCYPs* genes. *PgCYPs* belong to 9 clans were displayed in various colors.

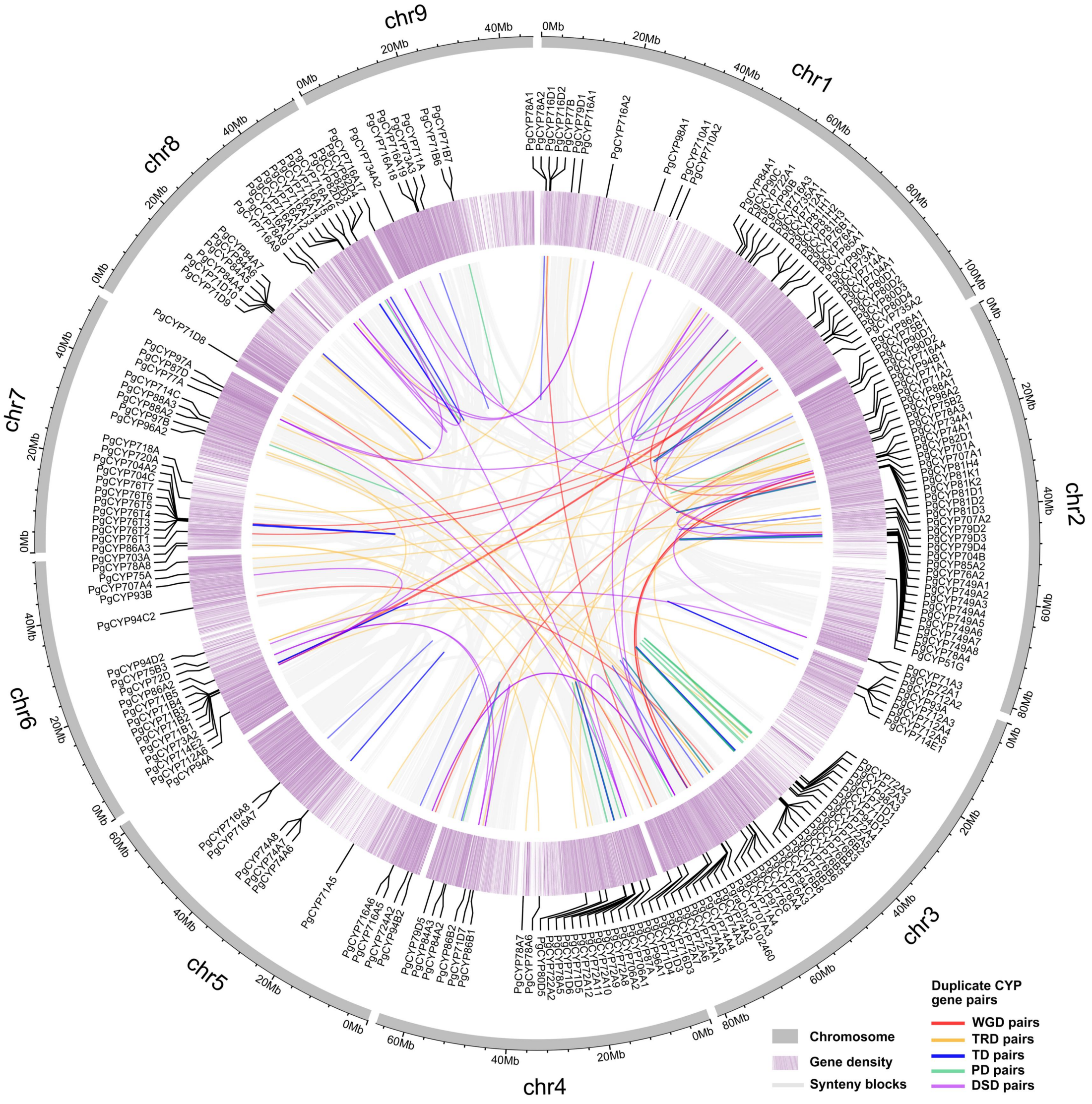

Figure S23. Duplicated gene pairs among CYPs. Light gray lines in the circle indicate all syntenic blocks in *P. grandiflorus*. The collineary analysis is performed by MCScanX. The inner track (light purple region) indicates gene density (number of genes per 0.1 Mb).

A

Motif	71 clan	85 clan	72 clan	86 clan	74 clan	97 clan	710 clan	711 clan	51 clan	Total
1	87	40	29	9		3		1		169
2	97	41	27	17	8	3	2	1	1	197
3	95	40	29	17	8	3	2	1	1	196
4	95	36	29	17		3	2		1	183
5	89	13	11			2		1		116
6	98			1						99
7	53									53
8	98	37		8				1	1	145
9	94	42	9	14	3	2	2			166
10	95	5				3				103
11	94	18	28	12		3	2	1		158
12			29							29
13	62	3	29	8		2		1		105
14	29	2		16		3				50
15	90									90

B

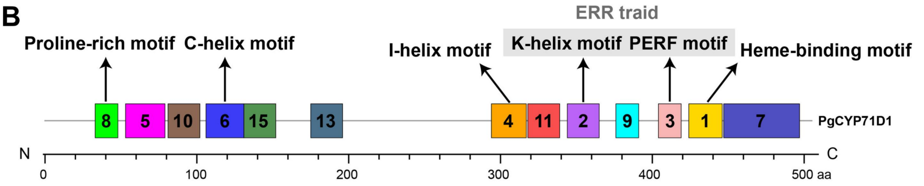

C

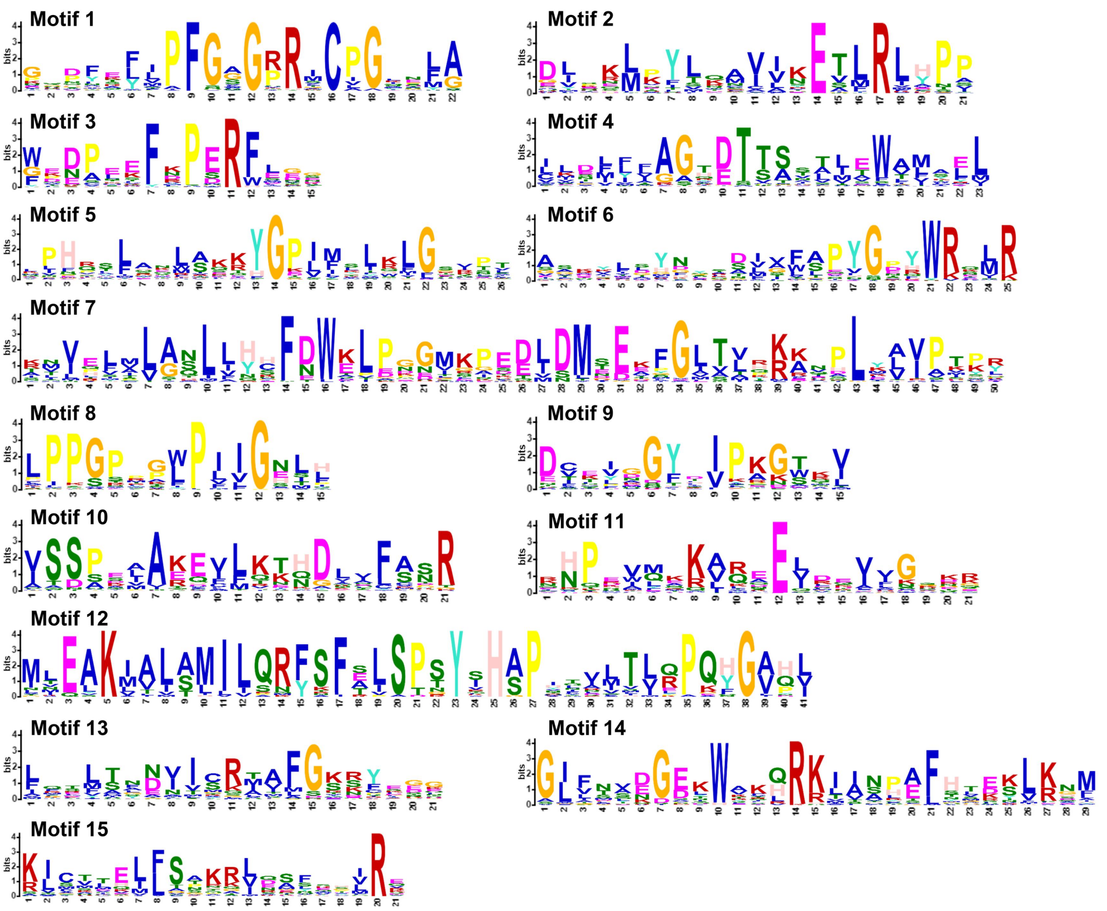

Figure S24. Distribution of conserved motifs in nine clans of *P. grandiflorus*. (A) Distribution of 15 conserved motifs in nine CYP clans. (B) A Schematic diagram of 15 conserved motifs in CYP proteins, taking PgCYP71D1 protein as an example, the signature motifs containing functionally characterized domains are indicated. N and C represent the N-terminal and C-terminal, respectively. (C) Protein sequences of 15 conserved motifs.

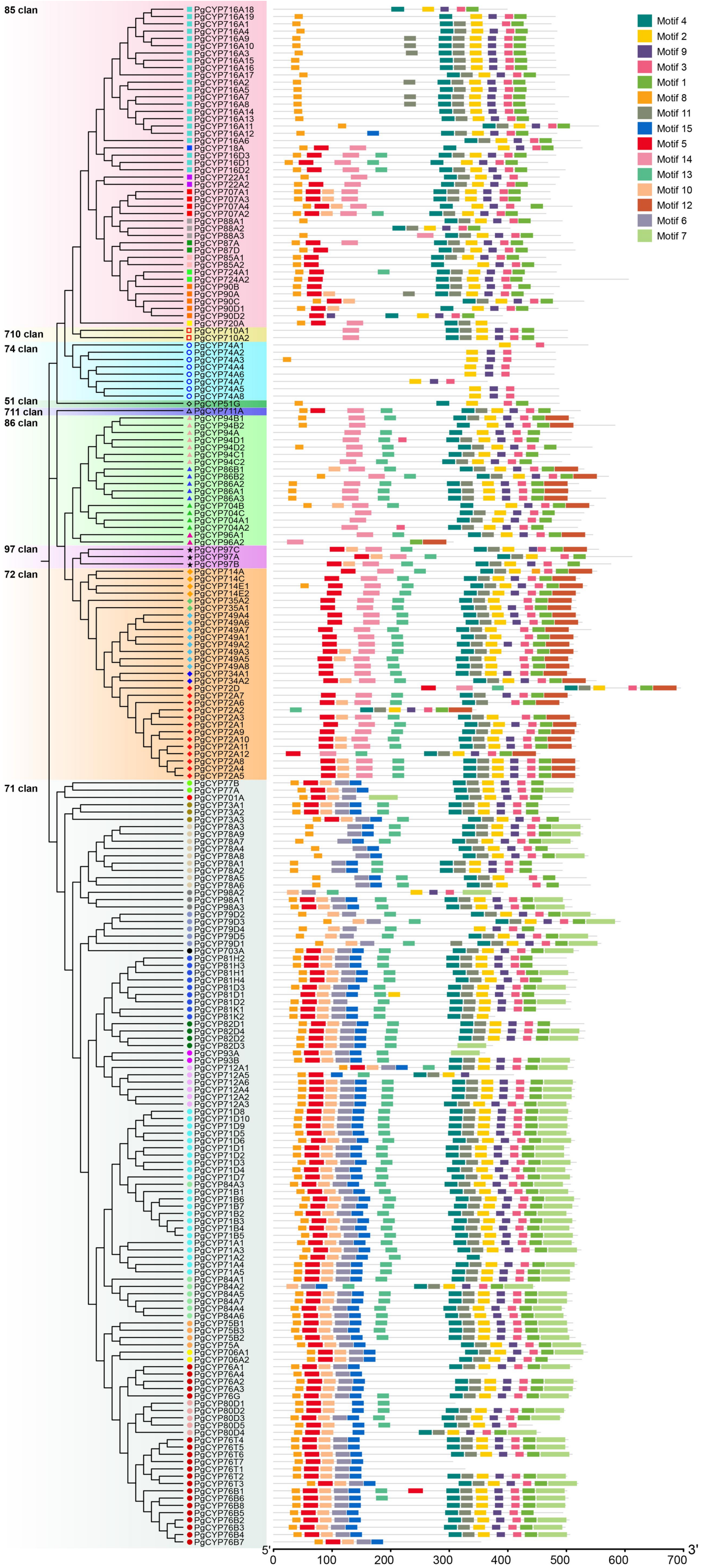

85 clan
710 clan
74 clan
51 clan
711 clan
86 clan
97 clan
72 clan
71 clan
Motif 4
Motif 2
Motif 9
Motif 3
Motif 1
Motif 8
Motif 11
Motif 15
Motif 5
Motif 14
Motif 13
Motif 10
Motif 12
Motif 6
Motif 7
5'
3'
0
100
200
300
400
500
600
700

Figure S25. Identification of conserved motif in 211 CYP proteins. The analysis was performed using MEME software.

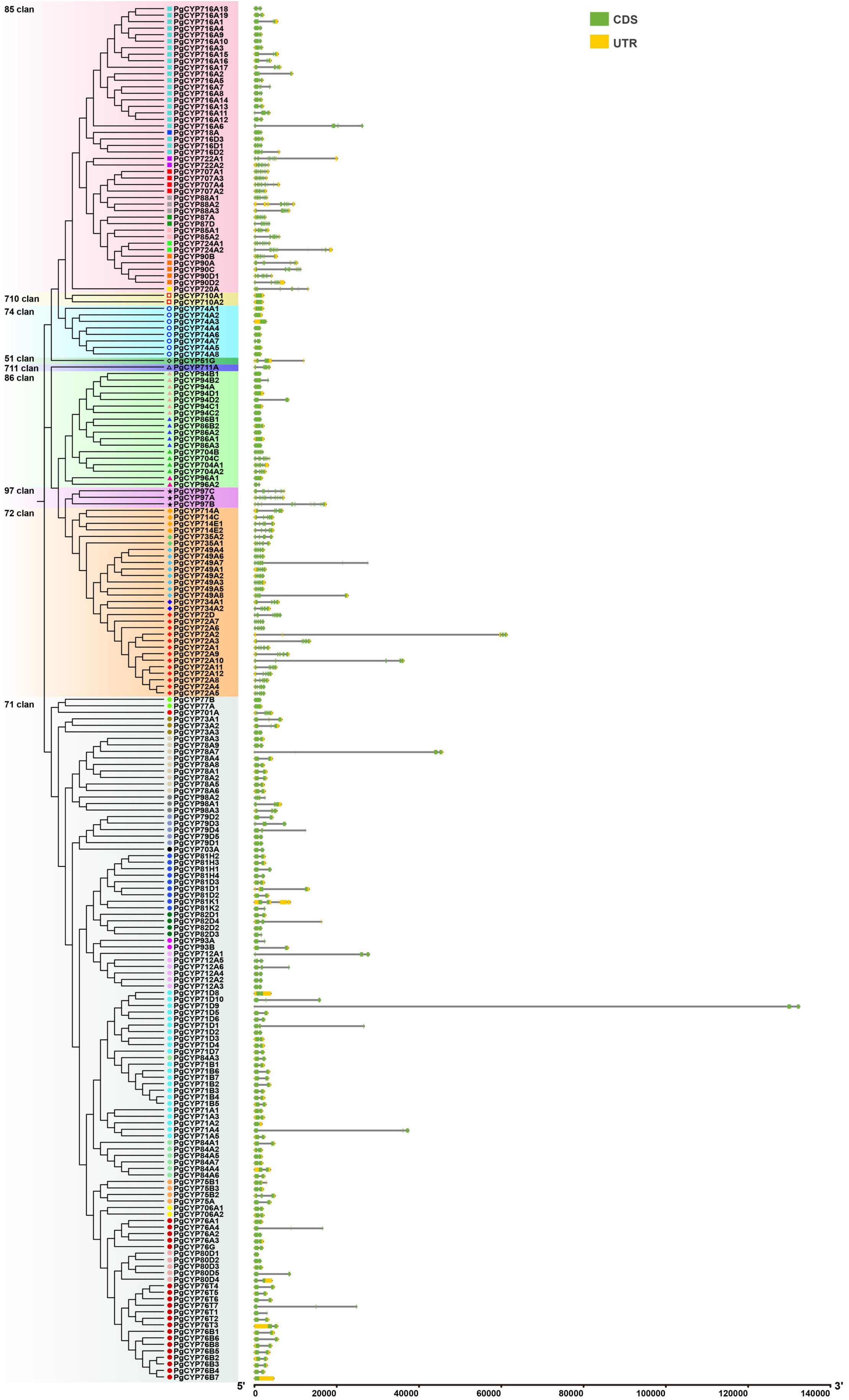
85 clan
710 clan
74 clan
51 clan
711 clan
86 clan
97 clan
72 clan
71 clan
CDS
UTR
5'
3'
0
20000
40000
60000
80000
100000
120000
140000

Figure S26. Gene structure of 211 CYPs. The structures were visualized using TBtools software. The green boxes and the yellow boxes represent the untranslated region (UTR) and coding sequence (CDS). The gray lines represent the introns.

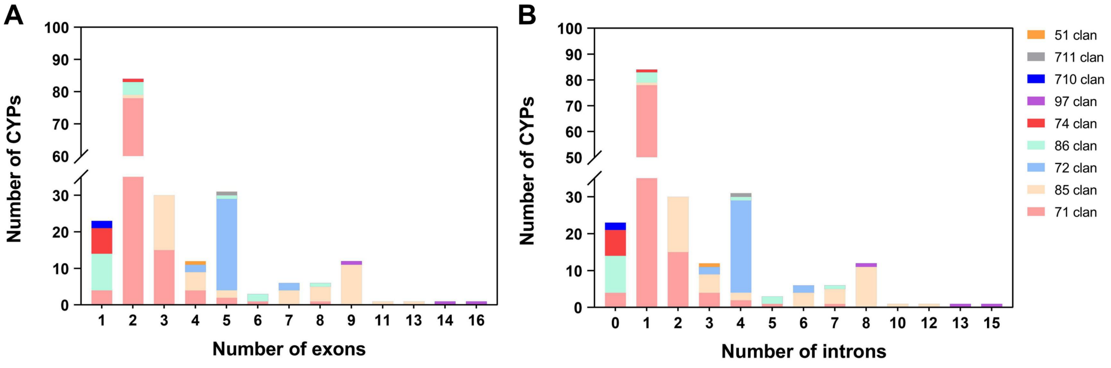

Figure S27. Distribution of exons (A) and introns (B) in nine clans of CYPs.

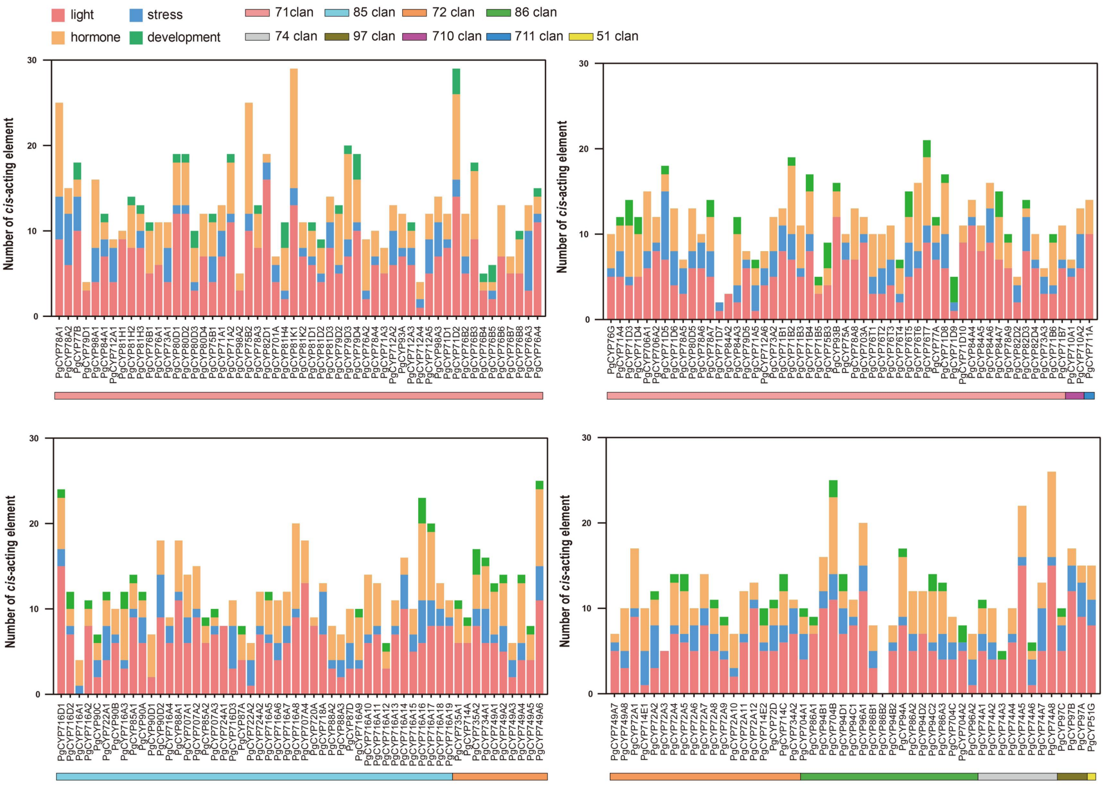

Figure S28. Distribution of *cis*-acting elements in the promoter region (upstream 2000 bp) of 211 CYPs. The predicted *cis*-acting elements were classified into development, light, stress, and hormone responsiveness.

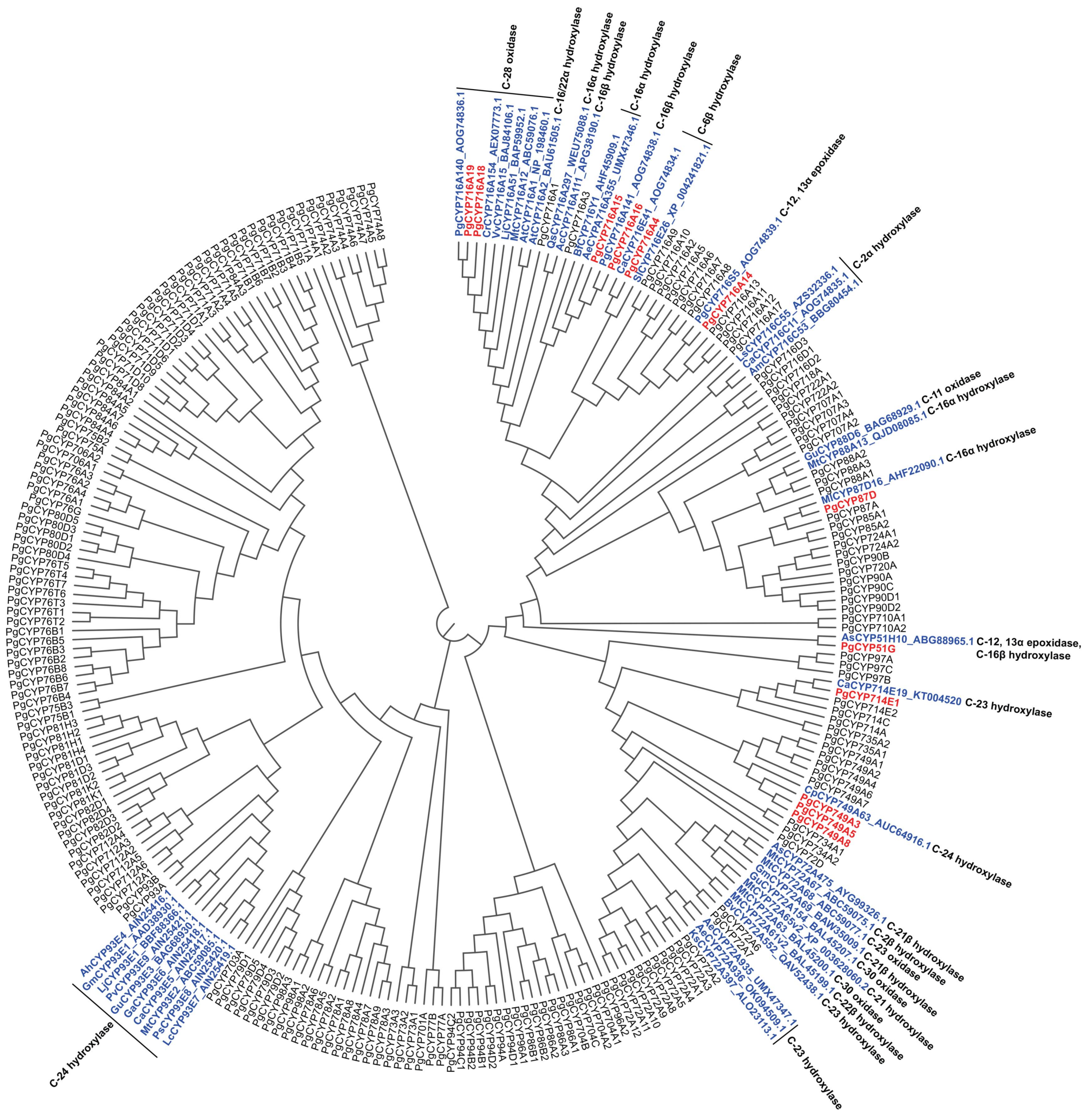

Figure S29. A maximum-likelihood tree of PgCYP proteins with functional CYPs in other species.
